# Follow the money: A startup-based measure of AI exposure across occupations, industries, and regions

**DOI:** 10.1093/pnasnexus/pgag185

**Published:** 2026-06-23

**Authors:** Enrico Maria Fenoaltea, Dario Mazzilli, Aurelio Patelli, Angelica Sbardella, Andrea Tacchella, Andrea Zaccaria, Marco Trombetti, Luciano Pietronero

**Affiliations:** Centro Ricerche Enrico Fermi (CREF), Via Panisperna 89 A, Rome 00184, Italy; Universitat de Barcelona Institute of Complex Systems (UBICS), Universitat de Barcelona, Barcelona 08028, Spain; Centro Ricerche Enrico Fermi (CREF), Via Panisperna 89 A, Rome 00184, Italy; Centro Ricerche Enrico Fermi (CREF), Via Panisperna 89 A, Rome 00184, Italy; Centro Ricerche Enrico Fermi (CREF), Via Panisperna 89 A, Rome 00184, Italy; Centro Ricerche Enrico Fermi (CREF), Via Panisperna 89 A, Rome 00184, Italy; Centro Ricerche Enrico Fermi (CREF), Via Panisperna 89 A, Rome 00184, Italy; ISC-CNR, Via dei Taurini 19, Rome 00185, Italy; Translated srl, Via Indonesia 23, Rome 00144, Italy; Centro Ricerche Enrico Fermi (CREF), Via Panisperna 89 A, Rome 00184, Italy

## Abstract

The integration of AI into the workplace is advancing rapidly, necessitating robust metrics to evaluate its tangible impact on the labor market. Existing measures of AI occupational exposure focus primarily on the theoretical potential of AI to substitute or complement human labor based on technical feasibility, offering limited insights into actual adoption. To address this gap, we introduce the AI Startup Exposure (AISE) index, a novel metric based on O*NET occupational descriptions and AI applications developed by venture backed startups worldwide. Our findings indicate that even though white-collar high-skilled occupations are theoretically highly exposed, they are heterogeneously targeted by AI startups. Roles involving routine organizational tasks, such as data analysis and office management, show significant exposure, while occupations involving tasks that are tied to ethical or high-stakes considerations—such as judges or surgeons—present lower AISE scores, despite technical feasibility for automation. Our approach challenges the conventional assumption that high-skilled jobs uniformly face high AI risks, highlighting instead societal desirability and market-oriented choices as critical determinants of AI exposure. Contrary to fears of widespread job displacement, our findings suggest that AI adoption will be gradual and shaped by social factors as much as the technical feasibility of AI applications. This framework provides a forward-looking tool for policymakers to monitor the evolving impact of AI and navigate a fast changing labor market landscape.

Significance statement AI stands as one of the most important technological advancements of our era, sparking debate over its impact: will AI replace a large share of human jobs or will its effects be more limited and selective? We analyze the types of AI products developed by highly funded startups and construct a novel measure that captures the extent to which occupations are concretely affected by emerging AI technologies. Our findings reveal that many occupations commonly considered highly exposed to AI are, in practice, less affected by existing AI products. Beyond market constraints, this pattern is also explained by the nature of these occupations: they often involve high levels of responsibility and ethically sensitive decision-making, which limit AI exposure. Overall, our results suggest that AI exposure is not an indiscriminate technological wave, but rather a targeted and selective process shaped by market incentives and social constraints.

## Introduction

The public and scholarly debate on the employment effects of the new wave of AI developments, especially since the deployment of generative AI, is highly polarized and presents contrasting views on their potential risks and benefits. While some fear a jobless future, others foresee job creation and complementarity between human and automated tasks, potentially leading to a productivity boost due to AI (1–4). Beyond the differing views, there is consensus among academics and policymakers that the current wave of AI fundamentally differs from previous technological shifts (5), distinguished by its unprecedented ability to mimic human reasoning and creativity across a wide range of applications. This unique capability to perform complex, nonroutine tasks, exemplified by large language models (LLMs) like OpenAI ChatGPT and Anthropic Claude, coupled with its rapid pace of improvement (6, 7) position AI as potentially one of the “most significant general-purpose technology of our era” (8), with far-reaching implications for the economy and heterogeneous effects across jobs, sectors, and countries (3, 9, 10).

As AI applications continue to emerge, extensive research has already focused on their impact on the labor market. There is growing evidence that AI is reshaping labor demand (11–14); however, the overall employment effects remain unclear due to the large uncertainty on how these rapidly evolving technical developments will be adopted and deployed (9, 11, 15). Empirical findings vary widely, from low disruption (16–18) to high displacement potential (19). Some studies show that AI displays complementary effects (18, 20) and drives productivity gains, particularly in high-skilled service occupations (1, 21–23), while others remain inconclusive about whether AI leads to complementarity or substitution (24, 25). Concerns have emerged about AI adoption outpacing the ability of the labor market to adapt (17), the quality of newly created jobs (15, 26), and the uneven impact of AI across sectors and regions (5). These effects vary depending on structural factors such as sectoral specialization, R&D capacity, productivity, and workforce skills (27, 28). These concerns are further reinforced by recent evidence on the impact of AI on workers (29), which shows that AI adoption can increase productivity for senior workers (30), while potentially leaving younger workers behind (31, 32).

Acemoglu et al. (33) and Autor et al. (34) argue that AI’s emphasis on automation, rather than augmenting human tasks, risks further stagnating productivity, wages, and labor demand, while deepening income inequality (as already observed in Ref. (35)). To mitigate these risks, they advocate for a human complementarity approach supported by an appropriate set of government policies (33, 34, 36). Moreover, the diminished worker voice due to AI powered monitoring and surveillance, the dominance of private actors in the AI race, and the absence of clear legislative frameworks have sparked broader concerns about AI societal impact (9, 15, 37, 38). These developments also heighten worker anxieties related to job security, diminished autonomy, and the perceived obsolescence of their skills (39, 40).

The lack of consensus on the labor market and the social effects of AI mirrors long-standing debates on technological change. The Neoclassical and Schumpeterian traditions propose distinct compensation mechanisms linking innovation to job creation or destruction, where the direction and magnitude of these effects depend on the type of innovation, and the outcomes differ between firms, sectors and the overall economy, leading to divergent empirical findings (41, 42). Nonetheless, despite the absence of a unified perspective, the literature increasingly and broadly acknowledges that, unlike previous ICT-based technological transformations, AI exposure is disproportionately concentrated among high-skilled white-collar occupations (19, 25, 43). AI in fact primarily targets clerical occupations, impacting both routine and nonroutine cognitive tasks, while manual, operational, and technical activities remain relatively less exposed (1, 44).

One of the primary methodological and conceptual challenges for understanding the impact of AI on labor markets is the empirical identification of occupational AI exposure. Building on the task framework pioneered by Autor et al. (45), several recent approaches estimate the average AI exposure of occupations based on the overlap between AI capabilities and occupational tasks or abilities (2, 17, 19, 24, 25, 46–48). Among these, the AI Occupational Exposure (AIOE) index proposed by Felten et al. (24, 25) is becoming a standard in the literature and serves as a benchmark for our analysis. More recently, AI-assisted approaches have emerged, using LLMs to assess occupational exposure, as demonstrated by Gmyrek et al. (18), who estimate task-level scores of occupational exposure to AI using ChatGPT-4, and Eloundou et al. (19), who combine expert opinions with ChatGPT-4 classifications to quantify the impact of generative pretrained transformers (GPTs) on the US labor market. Finally, Webb (43), Meindl et al. (49), Septiandri et al. (50), and Sousa and Sytsma (51) have proposed patent-based methods, employing natural language processing (NLP) techniques to directly quantify the similarity of AI patent texts and occupational descriptions.

In these works, AI exposure is generally determined by three main steps: (i) selection of a set of relevant AI applications—such as language modeling or image recognition—either arbitrarily or based on AI benchmarks;^a^ (ii) assessment of the potential for task or ability substitution in various occupations, leveraging detailed descriptions from the O*NET occupational database, via expert judgment, crowd-sourcing platforms, or NLP techniques; (iii) definition of the occupational AI exposure index as the share of the occupation’s bundle of tasks/abilities that AI technologies are capable of substituting.

All of these efforts, while extremely valuable, share some shortcomings. First, relying on expert or crowd-sourced evaluations of AI capabilities may lead to nonreproducible, subjective estimates. This is partially mitigated in approaches relying on AI patents, which are more quantitative and less subjective. However, a significant disadvantage is that patents may not cover most AI applications since, as many other software advancements, they are not often patented. Moreover, patents may not allow mapping the most recent advancements in AI as there is usually a lag between their filing date and the time at which they are observed in patent repositories (52). Second, by focusing on technical feasibility, all these indices, irrespective of how they are built, inherently measure *theoretical* and *potential* AI exposure, not *actual* adoption within firms and industries (28, 53). With the exception of Svanberg et al. (53), who take into account the *economic attractiveness* of automating computer vision, none of these approaches directly includes information on the economic viability and the social desirability of adopting AI systems. This may limit their predictive accuracy and usefulness in guiding policy planning. In fact, as the actual diffusion of AI is still in its early stages, no strong evidence of labor substitution seems to emerge for occupations considered most exposed to AI according to existing indices (13, 26, 27, 54–56).

To overcome the theoretical nature of AI exposure metrics and building on existing approaches in the literature, especially those relying on LLMs (18, 19), in the present article, we propose a novel occupational AI exposure index, the *Occupational AI Startup Exposure* (AISE). AISE aims at measuring the near-future, actual exposure of occupations by proxying AI innovations with AI applications developed by worldwide startups that received venture capital backing from the US-based venture capital (VC) firm and startup accelerator Y Combinator (YC),^b^ and relying on a large sample of funded European startups sourced from the EU-Startups Directory as a robustness check. In practice, AISE assigns an exposure score to each occupation by leveraging Meta Llama3 large language model to assess the similarity between O*NET job descriptions and the descriptions of the AI applications developed by startups, based on the information on startups present on the website of Y Combinator and the EU-Startups Directory. A key advantage of our methodology in constructing AISE is its full reproducibility, as it leverages an open-weight LLM that can be freely and locally executed. Therefore, our exposure index can be easily updated as new AI startups are financed, enabling near real-time tracking of AI investments to inform effective policy development.

According to AISE, the occupations displaying higher exposure are general office clerks, data scientists, computer and information systems managers, and market research analysts and marketing specialists. These roles typically involve programming, information processing, or organizational tasks that are increasingly targeted by AI startups. In contrast, athletes and sports competitors, judges, and pediatric surgeons present lower AISE scores. These occupations have more diversified skill sets and often involve tasks that are less amenable to AI automation due to physical, ethical, or high-stakes considerations. In agreement with the “reverse skill-bias” predicted by Acemoglu and Restrepo (57), our findings indicate that high-skilled, high-education jobs display the highest AI exposure, albeit with some interesting deviations from standard exposure indices.

When comparing our exposure ranking with that of Felten et al. (24), we observe that jobs with both low AIOE and AISE tend to be primarily composed of manual tasks. As AIOE increases, we detect a heterogeneous pattern, with several occupations displaying lower exposure from our startup-based index compared to the ability-based AIOE. These patterns can be explained by several factors; we identify two that play a particularly important role in shaping the use and impact of AI technologies across occupations: the simultaneous presence of multiple crucial skills and the ethical concerns related to the nature of the job. Interestingly, however, high-skilled occupations typically requiring a master’s degree or higher and significant experience, display high AIOE but lower AISE. This heterogeneity can help to disentangle jobs with similar abilities but different levels of actual AI exposure and suggests that, despite the theoretical exposure to AI, according to our startup-based approach many high-skill, high-education roles are not currently impacted by AI. This suggests that the necessity for advanced skills and the high-stakes associated with errors in these roles make AI integration less straightforward, even when there is theoretical potential for AI involvement.

Additionally, we propose a geographical and a sectoral projection of AISE. Geographically, our analysis shows that knowledge-intensive US metropolitan areas with expanding digital economies and tech industries, such as the Bay Area and Boston, display the highest average AISE, while regions more reliant on manufacturing or agriculture, particularly in the Midwest, exhibit lower exposure. Service-oriented industries that rely heavily on information processing and on high-skilled professionals are more exposed by AI; sectors like education and health care, which also require high education and training levels but involve several high-stakes jobs, exhibit intermediate exposure, while construction and agriculture are less frequently targeted by AI startups. Finally, a preliminary analysis on the exposure to AI and robotics integration shows that this type of AI-powered automation could drive a larger and widespread job disruption, also in manual occupations.

The novel measure we propose is grounded in actual investments in AI and provides a more realistic assessment of occupational exposure. Our findings in fact mitigate the high-skill catastrophe envisioned by other approaches, even though they suggest that the potential impact of AI robotics may be highly pervasive. Indeed, unlike the abstract nature of AI capabilities found in patent or benchmark datasets, AI startups are funded by venture capital because they propose tangible solutions related to the performance of specific tasks, prioritizing economic viability over potential technological feasibility and capturing societal interest, trust, and the willingness to integrate AI into occupations (58). A key advantage of the methodology we employ to construct AISE is its full reproducibility, as it leverages an open-weight LLM that can be freely and locally executed. Therefore, our exposure index can be easily updated as new AI startups are financed, enabling near real-time tracking of AI investments to inform effective policy development.

While we remain silent on the adoption patterns and net employment effects of AI innovation, we find important to stress that our approach is significantly closer to measuring adoption than others in the literature, especially those based on AI benchmarks. AISE reflects the market-screened expectations embedded in upstream VC investment decisions. Therefore, we do not frame it as a quantitative forecast of future adoption, nor a measure of current adoption and AI investment patterns,^c^ but as a source of complementary information that captures the innovation frontier and potential diffusion via startups and investments that might foreshadow adoption. In fact, VC investments rely on an implicit evaluation of market size, potential adoption, and thus commercial viability because the projects have passed rigorous investor due diligence screenings (with recent estimates placing Y Combinator acceptance rate around or even below 1): this is the essence of our *follow the money* approach.

## Results

### From AI startups to AI exposure

To quantify the AISE of occupations, geographical areas, and industries, we rely on two textual data sources: O*NET occupational descriptions and descriptions of startups sourced from Y Combinator and the EU-Startups Directory. We then link new AI developments with occupational characteristics using Meta LLM Llama 3. The choice of connecting two sets of unstructured descriptions using a LLM, rather than simpler NLP methods such as text mining or verb–noun similarity (as done, eg by Webb (43)), reflects an increasingly standard approach in contributions aimed at quantifying AI exposure (18, 19, 50) because LLMs better capture the semantic nuances in AI technologies and their link to the content of occupations. Although we rely on Llama 3 for its open-weight availability and the efficiency of its 8B version also with limited hardware, to ensure the full reproducibility of our results, in SI Section S6, we also show consistent results using OpenAI GPT-4o, which is a more powerful but closed-weight model accessible only through a paywall.

In practice, to build the AISE index, we feed Llama 3 with the textual short descriptions of (i) standard occupational classification (SOC) occupations provided by O*NET, and (ii) AI-tagged startups funded by Y Combinator (covering a global sample, but with the majority of startups being mostly based in the United States) and benchmark it with AI startups based in the EU, the United Kingdom, Switzerland, and Norway from the EU-Startups Directory. All selected startups underwent a competitive selection process and have secured at least 500,000 USD in funding. We then exploit the LLM linguistic abstraction capabilities to determine whether, for each startup-occupation pair, the startup’s AI application matches the O*NET short occupational descriptions (see Methods section for more details on the prompt strategy).

In the following, we present the results derived from Y Combinator startups, as they are funded by the same accelerator under the same funding conditions, resulting in a more homogeneous and higher-quality dataset. As shown in SI Section S9, the results obtained using the EU startup information are highly correlated with those based on Y Combinator, ensuring robustness and generalisability across the occupational labor markets in many advanced economies. We then define our Occupational AI Startup Exposure (Occupational AISE) for each job as the normalized number of startups developing AI applications matched by the LLM with the O*NET occupation short description, which provides a summary of the essential tasks associated with each occupation. Unlike other approaches in the literature that measure AI exposure as the share of tasks or abilities considered exposed to AI in each occupation (19, 25, 43), we chose to rely on O*NET short descriptions of occupations, because they better capture the core meaning and social context of a job. Our aim is not to identify which specific tasks can be automated, but rather to understand which occupations AI startups are engaging with. A discussion of this choice, along with an analysis showing that the results obtained using detailed work activities are consistent with those based on O*NET short descriptions, is provided in SI Section S12.

As mentioned in the Introduction section, AISE remains neutral on whether AI complements or substitutes for human labor in performing a job. Instead, it reflects its potential transformation driven by AI considering the influence on essential job tasks that, if replaced, may significantly alter the nature of the job and the skill-set required to perform it, hence indirectly capturing the AI application feasibility, cost, and attractiveness assessments by both startup and investors (53).

### Occupational AISE

To provide an initial impression of where the AI startup market is headed and which insights Occupational AISE can provide, we look at which jobs are most and least exposed to AI according to our analysis. An extended list of the most and least exposed occupations according to our index is presented in SI Section S1 (Tables S1 and S2).

The job with the highest Occupational AISE is *General office clerks*, described by O*NET as requiring *Knowledge of office systems and procedures* and which tasks include: “Maintain and update filing, inventory, mailing, and database systems, either manually or using a computer” and “Compile, copy, sort and file records of office activities, business transactions, and other activities.” In view of the rapid advancement of generative AI, which is increasingly simplifying and automating the generation and processing of any format of information (text, audio, and video), it is likely that a large portion of these tasks is highly substitutable by startups developing LLMs or AI agents. Among the AI applications developed by Y Combinator startups that affect the essential tasks of a general office clerk, we mention two illustrative cases: the startup *Nowadays* develops an AI-powered event planning copilot capable of organizing large-scale corporate events, eg contacting venues, negotiating, and handling administrative tasks; while *Quickchat AI* develops a platform to build multilingual AI assistants powered by generative AI models such as GPT that can perform conversational tasks, eg answering phone calls or processing information for organizational purposes. Other jobs with high Occupational AISE include *Data scientists*, *Computer and information systems managers*, and *Market research analysts and marketing specialists*. According to O*NET, all these occupations require tasks related to programming and information processing or organizational and planning tasks that, by the same token, are increasingly targeted by AI startups.

Analysing the jobs least exposed to AI according to Occupational AISE is less straightforward because they present more diversified skill-sets and educational/training requirements. To illustrate this, let us consider three jobs with low Occupational AISE scores: *Athletes and sports competitors*, *Magistrate judges*, and *Pediatric surgeons*. Athletes, while benefiting from AI tools for performance monitoring and injury prevention, primarily engage in tasks that rely on physical abilities and their societal value lies intrinsically in their ability to push human limits while remaining human. In contrast, judges perform tasks requiring advanced cognitive skills, such as information processing and decision-making in complex contexts, tasks that in principle generative AI could already perform. However, the high-stakes and ethically charged nature of judicial work poses significant barriers to AI adoption in this field. This reflects the heated debate about the role of AI in legal and judicial contexts (59, 60), the ethical implications, and the introduction of undesired biases in the increasing use of AI in the US criminal justice system (61–63). This highlights the critical role of the “human factor” and personal accountability in judges’ rulings, all elements contributing to a limitation to automation, beyond a mere assessment of technological feasibility. Finally, pediatric surgeons, like other medical professionals with low Occupational AISE scores, require a combination of manual skills (such as handling instruments or treating patients), cognitive abilities, and social skills. Although some essential tasks such as symptom-based diagnosis could be, and in some contexts already are, automated with AI (64, 65), the sensitivity to errors of the medical field discourages AI startups from targeting the substitution of critical medical tasks.

These examples show how our measure of Occupational AISE effectively captures not only the overlap between human and AI capabilities in performing specific tasks but also the societal attractiveness of such exposure (58). This insight is made possible by the use of data based on concrete AI applications, reflecting both the potential and the limitations of AI integration across various professions. Indeed, AI exposure is not solely driven by technical feasibility, as multiple societal constraints can accelerate, slow down, or even halt AI adoption.

### AISE and AIOE: comparing two different understanding of AI exposure

To validate and illustrate how our startup-based AI exposure method differs from existing approaches, we compare Occupational AISE with the AIOE index introduced by Felten et al. (24), one of the most widely used measures in the literature. By considering an occupation as a bundle of O*NET abilities (such as *Deductive reasoning* or *Negotiation*), to build AIOE the authors first quantifies the AI exposure of individual abilities through a crowd-sourced survey; second, they measure an occupation’s AI exposure as the average exposure of its required abilities. For more details on AIOE and its construction, see Methods section. Since abilities are abstract attributes that can be associated with different occupations, AIOE measures the theoretical or potential AI exposure of a job based on what current technologies can do, rather than its impact in real-world settings (66, 67).

**Figure 1:**
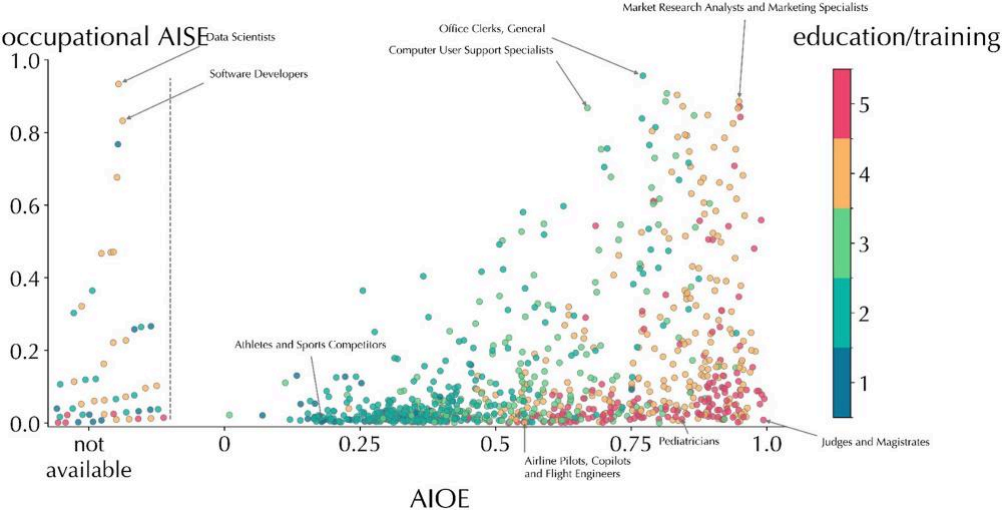
Occupation-level AISE versus AIOE scatter plot. Each dot represents a SOC occupation and is color-coded according to corresponding education and training level as described by O*NET job zones. The left column of points *not available* shows occupations for which no AIOE value is provided. Both indicators are normalized to range from 0 to 1, where lower values indicate lower levels of AI exposure.

Figure 1 shows the relationship between AISE and AIOE across SOC occupations. Each dot represents an occupation and is color-coded by its required level of education/training (indicated in O*NET as job zone). Occupations with education/training level 1 (shown in blue) require little or no preparation and rely mainly on manual skills, while those with education/training level 5 (shown in red) require extensive preparation and a larger share of cognitive skills. The first information we gather from Fig. 1 is that AIOE clearly ranks occupations assigning higher exposure to occupations with higher educational and training requirements, while for AISE the distribution of color is more mixed. Nevertheless, despite being based on very different methodologies, overall AIOE and AISE describe a coherent picture of AI exposure. In the bottom left portion of the plot, we find jobs composed mainly of manual and physical tasks, that are thus less targeted by AI-based startups and have been shown to be less subject to AI substitution (25). In contrast, as AIOE increases, we detect a heterogeneous pattern of Occupational AISE, that spans across levels of education and training, with several occupations displaying a lower exposure from AISE with respect to AIOE.

These differences are informative about the underlying factors the two exposure metrics capture. In fact, while we learn from AIOE which individual abilities required for a job could be affected by AI from a purely technological perspective, it does not account for other important factors, such as economic, social, or cultural constraints, that shape actual AI adoption. In contrast, Occupational AISE takes into account state-of-the-art AI applications and treats each occupation as a single unit of potential automation, capturing the societal and technical constraints that shape the development of AI applications aimed at substituting some or all of its essential tasks, thereby revealing a different dimension of exposure.

To illustrate, in O*NET *Database administrators* and *Lawyers* rely on similar cognitive abilities, such as *Deductive and Inductive Reasoning* or *Information Ordering*. As a result, AIOE assigns them nearly identical levels of potential AI exposure. However, according to our analysis, these two occupations are subject to very different degrees of exposure, with *Database administrators* and *Lawyers* displaying an Occupational AISE of about 0.8 and 0.05, respectively. No doubt, *Lawyers* and *Database administrators* differ for several reasons, especially linked to the societal implications of their professions: as discussed above, automating the judicial system presents both technical and ethical challenges, while there are fewer constraints on the administration of databases.

In the bottom right portion of the plot with high AIOE and low Occupational AISE, we find jobs such as high school teachers, judges, and marriage counselors. Even though AI can complement and support some secondary tasks of these jobs, our findings suggest that there is still no significant interest or trust in placing the essential tasks of these professions entirely in the hands of AI. However, it is important to mention that even within this region of the AISE–AIOE space, the reasons why certain occupations are not targeted by AI startups may differ, leading to distinct implications for AI adoption and impact. As illustrated above, two different cases may explain why some occupations, although technically feasible to automate, have low AISE values. In one case, the occupation involves critical skills and tasks with extremely low error tolerance (eg surgeons). In the other, substitution raises ethical concerns (eg judges). Clearly, the future trajectories of AI impact on these two types of occupations will differ: the first will depend largely on technical advancements that increase trust in delegating critical tasks to AI, while the second will be guided primarily by the ethical and cultural evolution of society.

Moreover, if we look at the distribution of color in the plot in more detail, overall high-skilled, high-education jobs appear to be the most at risk, in agreement with the literature (9, 11, 43). However, fundamental differences between the two indicators can be appreciated: AIOE clearly ranks occupations according to their education/training requirements, whereas for AISE the distribution of color is more mixed.

Higher AIOE maps into occupations relying mainly on problem-solving, logical reasoning, and information processing capabilities (24). Even so, occupations with education/training levels 4 and 5, which typically require a master’s degree or higher and significant experience, are concentrated in the bottom right of the scatter plot and display low Occupational AISE and high AIOE. This indicates that, despite their potential exposure to AI as signaled by AIOE, most of these high-education and high-experience roles are not currently targeted by AI startups.

**Figure 2:**
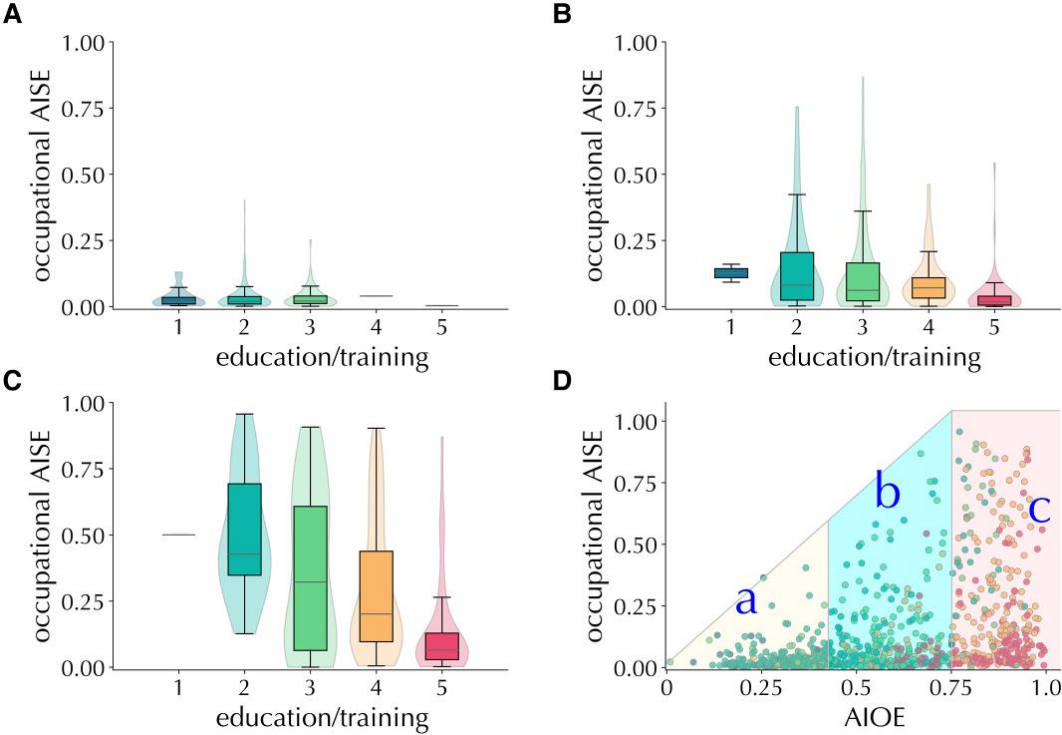
Occupational AISE by education and training level across AIOE ranges. a–c) Box–violin plots of occupational AISE values for increasing levels of education and training (according to the five O*NET job zones), corresponding to the AIOE ranges marked as *a*, *b*, and *c* in (d). Each box shows the interquartile range (from the 25th to the 75th percentile), with the central line indicating the median value of AISE; whiskers extend up to 1.5× the interquartile range. The overlaid violin plots illustrate the distribution of AISE within each group, highlighting the density of observations and potential outliers. d) The AISE–AIOE scatter plot (as in Fig. 1) divided into AIOE terciles, corresponding to the three areas shown in (a–c).

To further illustrate the key differences between the two exposure indicators, Fig. 2 divides the AISE–AIOE scatter plot into three equal-sized groups (terciles) based on AIOE values (Fig. 2d). Within each group, Panels a–c show box–violin plots of AISE by education and training level. In the lower AIOE tercile (Fig. 2a), AISE values are uniformly low across all levels of education/training. This suggests that in less AI-affected domains, differences in education and training requirements do not substantially influence occupational exposure. In the intermediate AIOE tercile (Fig. 2b), AISE levels rise moderately and begin to differentiate by education/training levels: higher-skill occupations (red boxes) show higher median exposure and greater variability, indicating that AI starts to interact more unevenly with task structures. Figure. 2c focuses on the upper AIOE tercile, capturing occupations with the highest theoretical exposure to AI. In this range, AISE values are overall higher, confirming that occupations with high AIOE are indeed more likely to be affected by AI. However, they are also more dispersed in terms of AISE, indicating that startup-based exposure among these occupations is both greater in magnitude and more heterogeneous in nature. The red boxes, corresponding to high-skill, high-education occupations, display the highest median exposure but also a wide spread, suggesting that, according to AISE, not all advanced occupations are equally affected by AI technologies. Conversely, occupations with lower education/training levels (blue and green) also exhibit relatively high AISE values, implying that certain mid- and low-skill occupations are increasingly integrated into AI-intensive domains.

This reinforces our previous argument that technological feasibility is not the sole factor driving the AI-based startup market. Even with a comparable level of theoretical exposure, jobs requiring more specialized or complex skills and education are less likely to see AI replacing humans in their essential tasks. Highly specialized professions require a combination of advanced education, extensive experience, and strong cognitive and social skills to handle uncertainty, such as that faced by judges or medical doctors. Traits that make the practical integration of AI less straightforward, even when technically feasible.

Although O*NET does not explicitly identify occupations involving uncertainty or ethical and health-related responsibilities, looking at the detailed skill composition of jobs with high AIOE helps reveal their qualitative differences. Each occupation in O*NET is associated with a set of skills, and to each skill is assigned an importance score from 1 (not important) to 5 (very important). In this analysis, we define as *crucial skills* those with importance scores >4.

**Figure 3:**
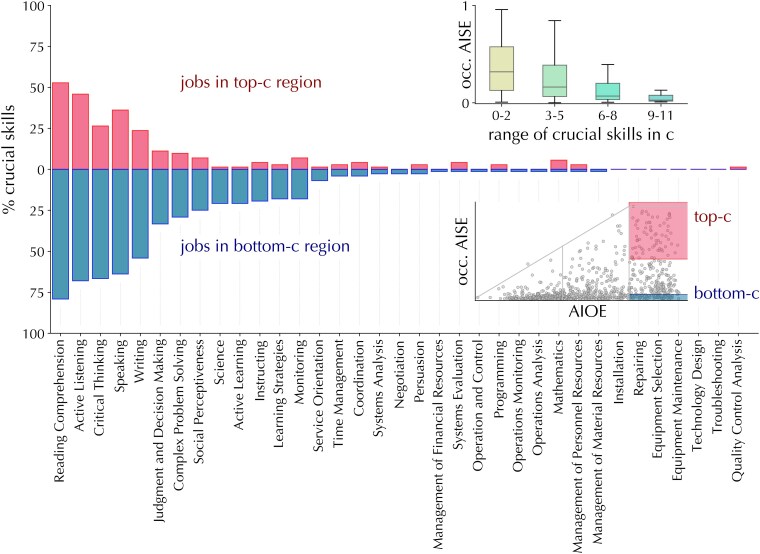
Distribution of crucial skills for occupations in the high-AIOE (“c”) region of Fig. 2. The main panel compares the frequency of crucial skills (skills with importance >4 in O*NET) for occupations located in the high-AIOE range (top AIOE tertile) distinguishing between those in the *top-c* region (higher AISE quartiles, bars above the horizontal line representing the 0% of crucial skills) and those in the *bottom-c* region (lower AISE quartiles, bars below the horizontal line representing the 0% of crucial skills). Bars indicate the percentage of occupations in each group for which a given skill is considered crucial. The bottom-right inset shows the AISE–AIOE diagram, highlighting the *top-c* and *bottom-c* areas within the c-region of Fig. 2. The top inset shows the barplot of the AISE values for different ranges of crucial skill presence in the occupations in the top c-region.

Figure 3 shows the number of high-AIOE occupations that require a specific crucial skill (on the x-axis). In particular, it focuses on the c quadrant of the AIOE–AISE diagram in Fig. 2 and highlights two groups, corresponding to the highest (top-c) and lowest (bottom-c) quartiles of AISE within the c quadrant, namely high AIOE-high AISE occupations and high AIOE-low AISE occupations, represented respectively in red and blue. Firstly, we observe that the likelihood of requiring crucial skills, including skills that generative AI can already perform reasonably well, such as *reading comprehension* and *writing*, is significantly higher in the *bottom-c* region, suggesting that in high-skill roles errors in task execution are costly and may slow down automation. Although *reading comprehension*, *active listening*, *writing*, and *critical thinking* are the most common crucial skills in both regions, in the *bottom-c* region the share of jobs requiring them is markedly higher. Especially *critical thinking* present in around 65% of jobs in the *bottom c* region versus the 25% in the *top-c* region. Secondly, in the *bottom-c* region, social and complex cognitive skills linked to decision making and problem solving—such as *judgment and decision making*, *complex problem solving*, *science*, *active learning*, *social perceptiveness*, and *instructing*—appear more frequently. These skills are crucial for managing uncertainty and present challenges to AI integration, even when there is high theoretical potential for AI involvement as proxied by high AIOE. More generally, the breadth of crucial skills appears to moderate exposure: at a given AIOE, occupations with more crucial skills tend to show lower Occupational AISE. The top inset of Fig. 3 illustrates this by presenting AISE distributions across four intervals of crucial skill counts for all high-AIOE occupations in the *c* region of Fig. 2.

While, as already mentioned, neither AIOE nor AISE explicitly distinguishes AI labor complementarity or substitution, the patterns we observe in the AISE–AIOE diagram, especially when looking at skills, suggest that combining these two metrics may provide useful insight into the nature of future AI exposure. It is reasonable to expect that occupations with high AIOE but low AISE (the bottom-c region in Fig. 2) are more likely to be complemented by AI rather than replaced—for example, through general-purpose tools that support secondary tasks rather than core activities. Conversely, occupations that score high on both AIOE and AISE (the top-c region in Fig. 2) may be considered more likely to face direct task substitution. To quantitatively validate this hypothesis, in SI Section S5, we compare our results with the complementarity metric introduced by Pizzinelli et al. (68), confirming that occupations with high AIOE and low AISE exhibit the highest potential for complementarity. However, it is important to notice that distinguishing complementarity from substitution remains an open and challenging task, in part because the notion of complementarity can have multiple interpretations (eg augmentation versus partial substitution) and the metric proposed by Pizzinelli et al. is one of the several attempts proposed in the literature to capture different aspects of complementarity (69–71).

Finally, to test a case that should be more aligned with a clear substitution and automation effect, in SI Section S8, we propose a preliminary AI Startup robotic exposure (RSE) index by restricting our focus only to the applications developed by AI-robotics startups—see the SI for more details on the methodology and visual representations. This case study builds on the long-standing body of literature on the labor-saving impact of robot automation (12, 72, 73), while extending it to the more recent strand of research on the impact of AI-robotics integration, which many expect to exert an even stronger labor saving impact than AI alone (54, 74). Considering only startups that are integrating AI software with robot hardware reshapes the exposure diagram: several occupations with low AISE scores actually display high robotic startup exposure, especially for occupations with lower education/training levels, which require more manual abilities or skills. High-skilled occupations generally remain low on both indices, but a smaller, albeit significant number of high-AISE jobs are also increasingly integrated with robotics. This suggests that the joint action of AI and robotics could transform occupations beyond manufacturing, including clerical and information processing roles.

### Geographical and sectoral AISE

**Figure 4:**
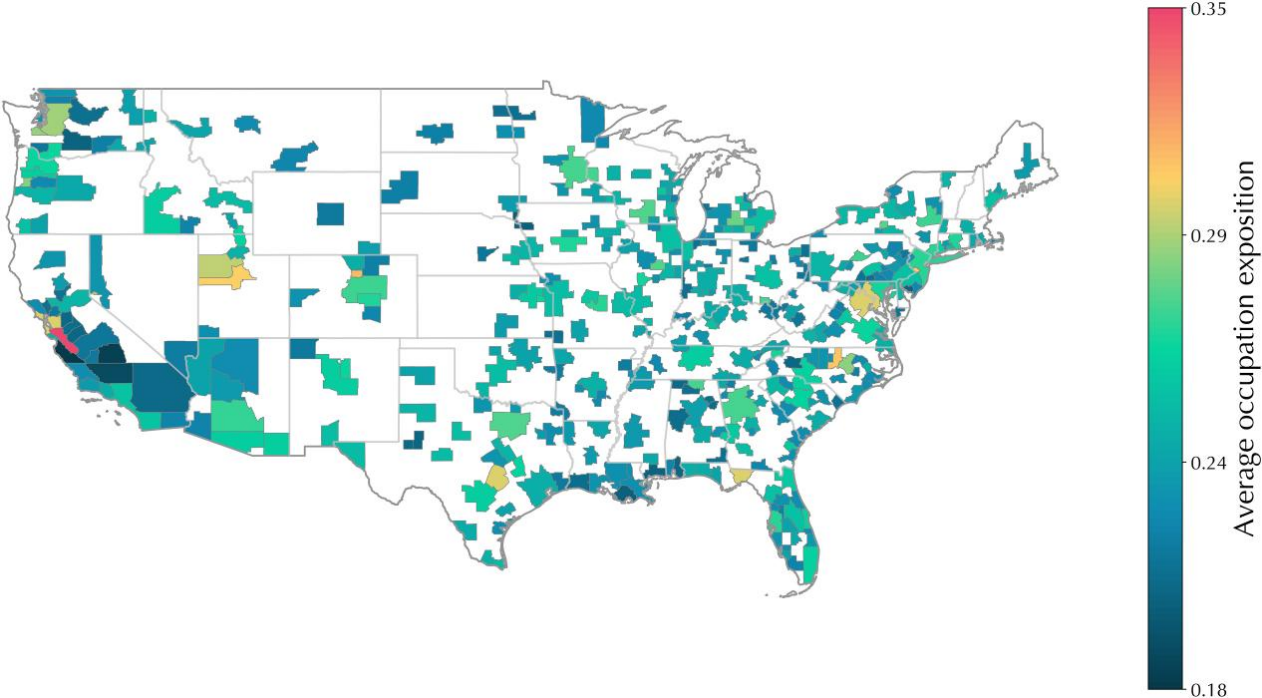
Geographical AISE. Map of average geographical AI startup exposure in US Metropolitan Statistical Areas.

Neither AIOE nor AISE directly measures the geographical dimension of AI exposure for the national or sub-national workforce. However, the net exposure at the geographical level can be assessed by calculating the average occupation exposure at different geographical scales (*Geographical AISE*). To this aim, we average Occupational AISE with the employment share per occupation in US Metropolitan Statistical Areas (MSAs), drawn from the US Bureau of Labor Statistics occupational employment figures,^d^ as detailed in Data section. Figure 4 shows that the Geographical AISE of US MSAs displays high geographical heterogeneity, highlighting how AI exposure of local workforces is unevenly distributed and is linked to local economic structures and path-dependent specialization profiles. According to AISE, only a few areas exhibit a high average exposure to AI (yellow–red), especially in regions with expanding digital economies, tech industries and innovation ecosystems, especially in the Silicon Valley (with San Jose/Santa Clara being the only MSA colored in red), the San Francisco Bay Area, and San Diego, with additional hotspots around Boston, Washington DC, Austin, Denver/Boulder, Salt Lake City/Provo, and Miami. Instead, metropolitan areas characterized by manufacturing and agricultural specialization, industries that have been slower to integrate AI technologies compared to high tech hubs, especially in the Midwest, show the lowest exposure to AI. This spatial distribution is consistent with Felten et al.’s geographic exposure index AIGE (24), which is positively associated with Geographic AISE, even though with some noticeable differences in the most exposed areas reflecting those observed at the occupation-level, as we show in the SI Section S7.

**Figure 5:**
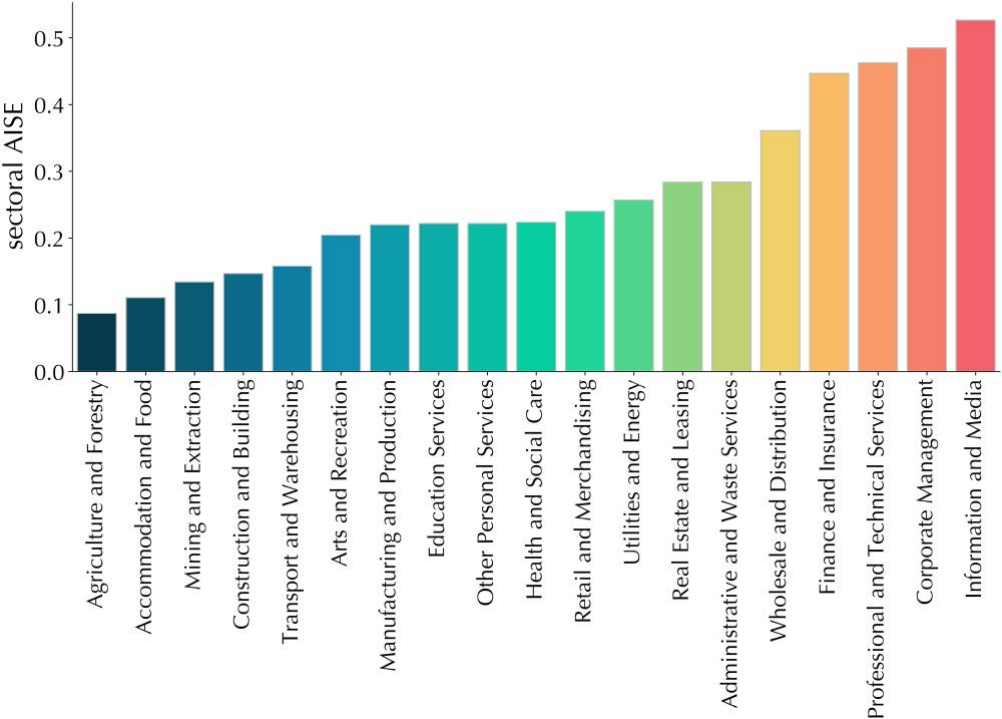
Sectoral AISE. Barplot of average sectoral AISE for two-digit NAICS industries in the US economy.

Similarly to the geographical exposure, we can also construct a measure of AI startup exposure at the sector level (*Sectoral AISE*) by computing a weighted average of occupational AISE across all occupations within a given industry, using occupational employment levels as weights. In particular, to build Fig. 5, we rely on US Bureau of Labor Statistics employment levels for two-digit NAICS industries, as detailed in Data section. Figure 5 displays two-digit NAICS sectors ranked in ascending sectoral AISE order.

The lowest-scoring sectors are those dominated by manual labor, such as construction, agriculture, food preparation and transportation, where tasks are physical, context-dependent, and difficult to substitute with software-only AI. Manufacturing, art and entertainment, educational services, retail trade, and real estate display intermediate exposure. In contrast, the most exposed sectors are predominantly service-oriented and information-intensive, where work relies on text- and data-heavy processes typically undertaken by workers with higher educational attainment (ie high job zones) and the corresponding occupations are placed in the right portion of the AISE–AIOE diagram (*c region* in Fig. 2d). Information, Management, Professional, Scientific and Technical Services, Finance and Insurance, Wholesale Trade, and Administrative and Support are the top exposed (yellow to red), consistent with AI comparative advantage in routine or semi-routine cognitive tasks (drafting, classification, forecasting, compliance, customer support). A broad middle (Real Estate and Leasing, Utilities, Retail, Health Care and Social Assistance, Other Services, Educational Services, Manufacturing) combines lower and higher skill sets, as well as AI-amenable office/process work (billing, scheduling, documentation, planning) with on-site, interpersonal, or ethically charged tasks and crucial skills that may be harder to substitute, as illustrated by the case of healthcare and education which include jobs located in the lower-right area (*bottom c*) of the AISE–AIOE diagram. To further investigate the differences and similarities between our approach and that of Felten et al., in SI Section S7, we document a positive association between Sectoral AISE and AIIE.

**Figure 6:**
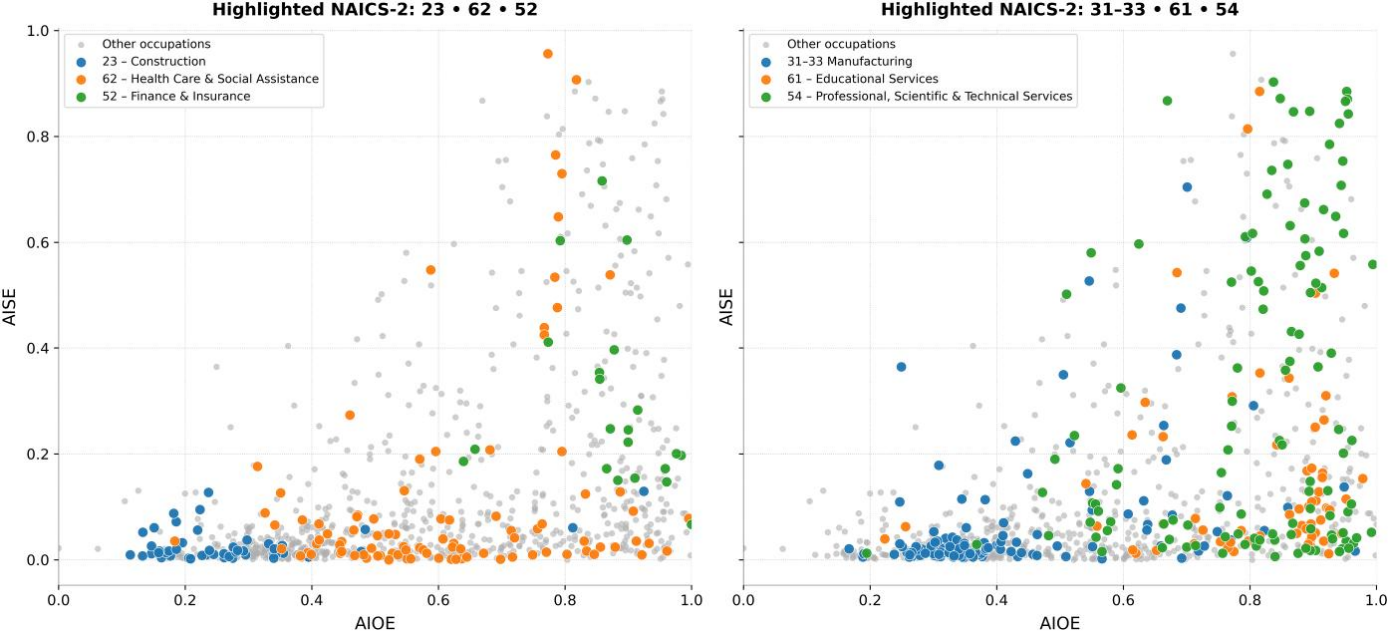
AISE–AIOE space for two-digit NAICS sectors. Each dot represents an occupation and is color-coded according to the NAICS sector in which the occupation is most represented.

To explore these patterns more closely, Fig. 6 displays the AISE–AIOE relationship, coloring each occupation by the two-digit NAICS sector in which it is most represented. Manual occupations in *Construction* and *Manufacturing* cluster in the lower-left portion of the plot. The *Healthcare and Social Assistance* sector is concentrated in the lower portion of the plot, with the exception of a few high-AISE occupations. In contrast, sectors such as *Finance & Insurance* and *Professional, Scientific & Technical Services* occupy the right-hand side of the diagram and span a broad range of AISE values. Together with the hypothesis presented in the previous section, ie that occupations with high AIOE but low AISE tend to exhibit complementary rather than substitutive exposure to AI, this representation can help identify whether complementarity or substitution effects are more pronounced across sectors. For instance, occupations in *Healthcare & Social Assistance* fall predominantly in a region associated with complementary exposure, while those in *Professional, Scientific & Technical Services* show a wider dispersion, suggesting that within this sector AI exposure is more occupation-specific.

## Discussion

This article introduces the AISE index, a novel measure of exposure to AI for occupations, industries, and geographies using information on venture-backed startups, an increasingly important segment of the AI ecosystem (6, 75, 76). AISE is built by comparing, via a large language model, unstructured textual descriptions of startups that propose AI applications with the description of the most important tasks in each occupation.

By focusing on actual innovations developed by AI startups that have attracted significant funding from reliable investors, AISE allows us to map where entrepreneurial activity and investment currently intersect with the employment landscape. Adding the viewpoint of investors, albeit limited to venture capital, this perspective allows us to move from the question “which jobs might be impacted?” to the more immediate one of “which jobs are currently targeted by investors?.” This provides a complementary view to existing approaches by capturing not only where AI may be deployed based on current technical capabilities but also where the market is effectively heading, reflecting the economic viability as well as the societal and ethical factors that shape the real-world implementation and adoption of recent AI developments. Our approach overcomes some limitations of patent-based indicators, affected by time lags and the exclusion of nonpatentable applications, and of crowd-sourced or benchmark-based measures, which often yield speculative feasibility-driven assessments. It also allows for continuous refinement and expansion as new AI startups emerge, thereby potentially enabling the real-time tracking of fast-evolving AI innovations.

Our findings reveal a more nuanced pattern of occupational exposure to AI than commonly suggested in the literature, indicating that even among high-skilled white-collar professions, the impact of AI is not uniform, and meaningful differences emerge when examining each profession in its specificity. AISE assigns higher exposure to occupations involving routine tasks, such as office clerks, and data-intensive activities, such as data scientists and market research analysts, while manual, highly interpersonal, or ethically constrained professions appear considerably less exposed. Comparing AISE with the benchmark-based AIOE by Felten et al. (24) highlights the distinction between technical feasibility and market viability: although the two indicators are broadly aligned, AISE shows that occupations with higher education and training levels are less frequently targeted by startups, despite being theoretically highly exposed. For instance, judges and pediatric surgeons show high AIOE scores, indicating that a significant portion of their tasks could theoretically be performed by AI, yet they display low AISE.

This suggests that factors such as professional expertise, ethical responsibility, and uncertainty management still limit the practical substitution of complex human tasks by AI, particularly in high-stakes domains. In particular, we highlight two types of occupations where, despite technical feasibility, AI startups show limited interest: jobs requiring multiple crucial skills, especially when entailing critical or error-intolerant decision making, and those involving ethically sensitive judgment where human agency remains essential.

While our focus is limited to measuring exposure and a full assessment of AI impact on employment lies beyond the scope of this study, we provide two exploratory analyses in this direction. First, we further characterize occupations within the AISE–AIOE space, offering preliminary evidence on which occupations and sectors are more likely to experience a substitutive rather than complementary impact of AI. Second, a preliminary Robotic Startup Exposure index suggests that the synergy between AI software and robotic hardware may profoundly broaden the scope of automation, increasingly affecting also manual occupations. This robotics exercise is also of interest because it is one of the few cases in which we can isolate a specific type of AI application. In fact, existing exposure indices, and AISE is no exception, do not allow a systematic analysis of distinct AI domains. This is a nontrivial limitation as different types of technologies may exert very different effects on the labor market, and taking into account technological heterogeneity will be fundamental for understanding the nature and implications of AI exposure.

Extending the analysis across regions and sectors, AISE sheds light on a geographically and industrially heterogeneous landscape. US metropolitan areas with knowledge intensive economies and dense innovation ecosystems—such as the Bay Area, Boston, Austin, and Washington D.C.—face the highest exposure, while areas characterized by strong manufacturing and agricultural bases, especially in the Midwest, exhibit substantially lower exposure. Similarly, information, finance, and professional services emerge as the most exposed industries; manufacturing, educational services, and healthcare show comparatively lower exposure—potentially signaling complementary rather than substitutive AI adoption; finally, construction, mining, and agriculture exhibit the lowest exposure scores.

Our approach provides valuable insights by capturing how AI is being commercialized and attracting investment, however several limitations warrant consideration. First, the reliance on startup data introduces a bias toward innovations that are already commercially viable or have secured VC backing. This may lead to an underestimation of AI developments emerging from academia or from industries less dependent on venture capital. Moreover, startups alone may not capture the full scope of commercially viable AI applications, as many innovations in the AI domain are also carried out by public entities and are especially developed internally by big tech companies. To remedy this and obtain a more comprehensive picture of AI innovation, in future research we plan to use and integrate additional data sources beyond startups. However, it is worth noting that startups tend to develop specialized, domain-targeted technologies, whereas large companies focus on more general-purpose AI platforms (77, 78). Startups also often rely on infrastructures provided by big tech firms (eg models or cloud platforms) (79), and several major AI advances have originated in startups that later went through big tech acquisitions (eg *Scale AI*, *Play AI* both funded by Y Combinator, or, on a larger scale, *DeepMind Technologies*). Thus, while these limitations remain, analysing startups is a meaningful lens for capturing the broader technological dynamics within the AI ecosystem and how these are diffusing into specific occupations.

Second, as detailed in the Introduction section, AISE is an exposure indicator and therefore does not incorporate information on actual adoption patterns. Capturing this additional dimension would require, in future work, data on AI investments and/or employer–employee datasets that would allow to study the changing employment structure of adopting firms. At present, however, this is still challenging: diffusion and deployment are still at an early stage, evidence from recent survey-based studies suggests that actual large-scale AI adoption is still limited, around 6 of US and French firms, and concentrated in few technology-intensive sectors (notably, information, professional-scientific, and technical services sectors), among larger firms and in VC backed startups and firms (6, 75, 76). This is why currently the majority of occupational AI indices focus on exposure rather than impact. These patterns also support our focus on venture backed startups as a leading edge indicator of AI exposure and potential diffusion, one that is positioned closer to actual use than AI benchmarks alone. However, we acknowledge the limitation that our measure neither replaces comprehensive adoption surveys nor captures AI innovation across the full universe of firms. Instead, it focuses on a central locus where both innovation and adoption occur.

Additionally, by focusing on the occupational dimension, our index by design operates the implicit assumption that occupations are homogeneous across geography and across firms. However, this is not always the case, occupational heterogeneity can stem from the significant portion of AI-related tasks driven by the tacit knowledge embedded in firm-specific organizational practices and procedural routines (80–82), or from different labor market contexts (83). This makes AI exposure far more complex than occupational-level analysis can reflect (48). Again, to address these firm-level dynamics and for a more comprehensive and context-specific understanding of AI employment effects, a more granular approach beyond occupational-level exposure and more focused on firm-level capabilities and adoption would be necessary.

Looking ahead, while this study remains deliberately agnostic on the interpretation of geographical and sectoral exposure patterns, we plan to investigate the labor market implications of our exposure indices, examining how differential AI exposure across regions and sectors translates into wage and employment dynamics, as well as inequality. Furthermore, given the fast-moving nature of AI technologies, the results presented here should be viewed as a first step in an evolving analysis, as new breakthroughs and emerging trends are likely to reshape the innovation landscape and thus AI exposure. To capture these dynamics, in future research we plan to leverage the expanding information on AI startups to develop a dynamic version of the AISE index able to capture the exposure to different AI waves—as in the preliminary analysis presented in the SI.

We also aim at providing a more detailed picture of the evolution of AI innovation by more precisely quantifying the real-world AI innovation frontier through the inclusion of larger datasets from other startup ecosystems beyond Y Combinator and EU startups and the integration of startup data with additional sources such as AI patents, scientific publications, and codebases. By building on this, we also aim at deepening our understanding of whether new AI technologies primarily complements or substitutes human labor, incorporating new information on task-level substitution dynamics, following recent survey-based approaches for investigating AI adoption and its impact on specific tasks (84, 85). In this regard, we also plan to extend our analysis on the integration of AI in robotics—an area more closely associated with clear substitution mechanisms—as illustrated by the preliminary results presented in the SI.

In conclusion, this study offers a new perspective on AI labor market implications by grounding its analysis in market-driven innovations and, contrary to widespread fears of imminent job displacement in high-skilled occupations, it suggests that automation will target routine, economically viable tasks first, while expert-driven or ethically constrained roles will remain relatively shielded from AI disruption, at least in the short term. Our analysis, and the continuous refinement of the AISE index via incorporating diverse textual sources on AI advancements and tracking the evolving effects of AI, may provide policymakers with an effective tool to better anticipate labor market trends and develop informed strategies to address the challenges and opportunities presented by this new wave of technological change.

## Data and methods

### Data

#### Occupational and employment data

To characterize occupations, we rely on O*NET which is maintained by the US Department of Labor Employment and Training Administration.^e^ O*NET provides survey-based information about the skills, knowledge, tasks, tools, technologies, and educational requirements of each occupation, organized according to the O*NET-SOC occupational classification (86). In our analysis, we employ the O*NET-SOC lowest level of aggregation, thereby extracting information on 1,016 jobs and we employ three sets of occupation-level information: tasks, skills, education and training requirements (Job Zones).

First, and crucial to our methodology, to connect jobs to startup AI advancements, we consider the short description of each occupation, ie a summary of its essential tasks. We purposely use these short descriptions rather than the full task lists, because they effectively report the salient characteristics of the job while being concise, an essential feature for our analysis since we use these descriptions as input for our queries to the large language model Llama 3. For instance, the job description for Cardiologists reads: “Diagnose, treat, manage, and prevent diseases or conditions of the cardiovascular system. May further subspecialize in interventional procedures (eg balloon angioplasty and stent placement), echocardiography, or electrophysiology.” Feeding Llama3 with the description of an occupation that summarizes its essential tasks in two or three sentences, as opposed to the description of all the tasks that compose it, has the advantage of reducing noise because the complete task lists can be extensive and include detailed, context-specific tasks that are not central to the primary scope of the job. Therefore, this reduces the risk of overloading the language model with excessive detail while maintaining sufficient information about the essential features of each occupation. Second, we use occupational skills. O*NET defines a set of 35 skills (such as *writing, reading comprehension*, or *coordination*) that are associated with each occupation with an importance score ranging from 1 (not necessary) to 5 (essential). Third, we retrieve information on educational, experience, and on-the-job training requirements as described by O*NET job zones, and group occupations based on their similarity in human capital requirements. For instance, a job in job zone 1 requires little or no preparation, while a job in job zone 5 requires extensive preparation.^f^

Finally, to construct the sectoral and geographical AISE indices, we retrieve data about employment levels by SOC occupation and US Metropolitan Statistical Areas, as well as US-wide employment levels by two-digit NAICS industry from the *Quarterly Census of Employment and Wages* (QCEW) dataset maintained by the US *Bureau of Labor Statistics*.^g^

#### AI startup data

To obtain information on AI-innovative startups, we rely on the set of startups financed by the US-based technology startup accelerator and venture capital firm Y Combinator (YC), obtained from the official website of YC.^h^ The platform contains information on over 5,000 startups funded since YC’s launch in 2005, all of which have received a fixed amount of $500,000. For each startup, we extract its name, a brief and a detailed description, the YC funding year, and a set of thematic tags defined by YC. To construct our exposure index, we select only the startups with AI-related tags and use their detailed descriptions as input for queries to Llama 3. More specifically, we consider the following tags: *AI, artificial intelligence, AI-assistant, AI-powered drug discovery, AIOps, conversational AI, ML, machine learning, deep learning, deepfake detection, generative AI, AI-enhanced learning*, and *computer vision*. Thus, we obtain a subset of 958 AI startups funded by YC between 2005 and March 2024. More than 50 of these AI startups were funded after 2020; however, as shown in SI Section S10, the results obtained using all startups are highly correlated with those obtained using only the startups founded after 2020.

To test the robustness of our results based on the information sourced from Y Combinator, we also draw information on AI startups from the EU-Startups Directory, a curated database of over 30,000 startups based in Europe, the United Kingdom or Switzerland, that provides company-level information such as name, country, founding year, sector tags, and funding status.^i^ Additional details on the robustness procedure are provided in SI Section S9 From this dataset, after filtering by AI tags and funding level, we obtain a sample of 830 AI startups, founded between 2005 and 2024 (again, with the majority established in the past 10 years).

### Methods

#### LLM approach to quantify AISE

To construct our AI occupational exposure index, we select all the descriptions of funded AI-tagged startups and feed them to the large language model Llama 3 to infer if they developed/are developing a product or a service with the potential to replace some or all tasks associated to a job. For efficient and reproducible execution, we use the pretrained 8B version of Llama 3, ie the open-weight LLM released by Meta in April 2024.^j^ For each O*NET-SOC occupation, we iterate over all AI-tagged startups and feed the following prompt to Llama 3:

“role”: “system,” “content”: “You are an AI specialist.”

“role”: “user,” “content”: “Given the following startup description: ” + startup[j] + “and given the following job description: “+ job[i] + “can the product or service developed by the startup help directly replace humans in performing some of the described job’s tasks? Use only the information provided by the two descriptions. Reply only yes or no.”^k^

Where startup[j] represents the detailed description of startup *j* as obtained from the Y Combinator website, and job[i] the short description of job *i* provided by O*NET. The AI exposure of each occupation is then calculated as the number of startups for which Llama 3 responds *yes* normalized by the number of AI-tagged startups considered.

It is important to bear in mind that, although our prompt asks the LLM whether the startup can help replace some essential tasks, our measure is not a measure of substitutability. In fact, to obtain an affirmative (yes) response to our prompt, it is sufficient that a single task is deemed replaceable by Llama 3. Therefore, our index does not aim to quantify the potential labor-saving impact of AI. Instead, it should be interpreted as a measure of occupational AI exposure, reflecting the interest of the startup ecosystem in broadly influencing or interacting with a particular job in light of its social role and context.

Finally, we manually validate a subsample of Llama 3 outputs and find that the model achieves an accuracy of 95. Accuracy may be further improved by applying more sophisticated techniques (eg fine-tuning the model or training a classifier on the manually labeled examples), however, we deem a 95 accuracy as sufficient to support our main claims.^l^ Moreover, in the SI Section S6, we test the robustness of our methodology and results by repeating the experiment with a different input, LLM, and prompt.

#### Crowdsourced approach to quantify AIOE

To test and validate our measure of startup-based AI exposure, we compare Occupational AISE with a standard measure in the literature, the AIOE index introduced by Felten et al. (24). AIOE connects 10 AI applications sourced from the Electronic Frontier Foundation AI Progress Measurement project, such as image recognition or text generation, with 52 O*NET occupational abilities, such as *oral comprehension* and *inductive reasoning*. The AI application-ability degree of relatedness is established through a matrix crowdsourced from Amazon Mechanical Turk provided in Felten et al. (24), and each ability exposure is defined as the sum of its relatedness scores with the AI applications. The AIOE of each occupation is then computed as the weighted average of the exposures of the abilities used within the occupation. The weights are derived from the O*NET ability *level* (ranging from 1 to 7) which indicates the degree to which an ability is required to perform the job tasks, and the ability *importance* (ranging from 1 to 5) which indicates how critical the ability is for performing the job tasks. Since occupational characteristics are periodically updated in O*NET, to build the AIOE measure employed in this article, we draw the ability level and importance from O*NET 2024, which slightly differs from the version of O*NET used by Felten et al. Therefore, AIOE for each job *i* is computed as follows:


$${\text{AIOE}}_{i}=\frac{\sum_{\;j=1}^{52}A_{kj}L_{ij}I_{ij}}{\sum_{\;j=1}^{52}L_{ij}I_{ij}},$$

where *k* represents the AI application, *j* the ability, and $A_{kj}$ the exposure to AI of ability *j*. O*NET provides occupation-ability connections for 873 jobs. Therefore, we compare our exposure index with the AIOE only for these 873 jobs, a subset of the 1,016 jobs we use in our analysis.

While our index measures exposure by linking AI applications developed by startups directly to O*NET occupational descriptions, AIOE builds an occupation’s score as a weighted average of ability-level exposure and is therefore less occupation-specific (67). Therefore, by focusing solely on technological feasibility when identifying which abilities are more or less likely to be performed by AI and considering occupations not as a whole but as bundles of abilities with different exposure levels, AIOE is a forward looking measure of *potential* rather than *actual* AI exposure (24, 66). This means that a job requiring many abilities highly exposed to AI is only potentially exposed, since other factors, not explicitly covered by the sole ability dimension, may be at play and may mitigate or worsen its exposure, as we extensively argue in the Results section. Moreover, upon inspecting the abilities in O*NET, we note that they are not highly granular when it comes to cognitive abilities. For example, the ability *Written Expression* encompasses various writing skills, some of which—such as summarizing texts and writing reports—are already mastered at high levels by AI (particularly by modern language models), while in other areas, such as creative writing, AI still lags behind. As a result, jobs requiring creative writing should display lower exposure.

## Supplementary Material

pgag185_Supplementary_Data

## Data Availability

All data used in this article is publicly available. O*NET can be accessed at: www.onetcenter.org. Information on Y Combinator startups can be found at: https://www.ycombinator.com/. Information on EU startups is available at: https://www.eu-startups.com/. The US Bureau of Labor Statistics Quarterly Census of Employment and Wages is available at: https://www.bls.gov/cew/. The AI Occupational Exposure (AIOE) scores are available at: https://github.com/AIOE-Data/AIOE. AI Startup Exposure (AISE) data can be accessed at: https://github.com/AISE-Data/AISE.

## Notes

a AI benchmarks are standardized tests designed to evaluate AI performance in relevant domains such as visual reasoning and reading comprehension.

b Y Combinator, founded in 2005, is a leading startup accelerator that has funded over 4,000 startups, with a total combined valuation exceeding $600 billion. YC typically invests $500,000 in exchange for 7% equity, running two batches each year that last for 3 months. YC is renowned for its vast alumni network and mentorship programme and yielded more than 50 unicorn companies, including Airbnb, Dropbox, and Twitch.

c In no way do we argue that AISE is a quantitative forecast of future adoption, nor a measure of current adoption in specific sectors of the economy, particularly large employers such as the public sector.

d MSAs groups a core city and its surrounding areas that are tightly linked by commuting, so they capture economically integrated local labor markets more accurately than county boundaries. However, for completeness, in SI Section S7 (Fig. 10), we show the average Occupational AISE of US counties.

e The United States Occupational Information Network O*NET is available at: www.onetcenter.org.

f More details on O*NET job zones can be found at: www.onetonline.org/help/online/zones.

g The Quarterly Census of Employment and Wages (QCEW) dataset is available at: https://www.bls.gov/cew/.

h The description of Y Combinator backed startups is available at: www.ycombinator.com/companies.

i The EU-Startups Directory is available at: https://www.eu-startups.com/directory/.

j For more information on Llama 3, see https://ai.meta.com/blog/meta-Llama-3/.

k The prompt was optimized using a human–performance feedback loop to maximize agreement with human judgment. While AI can also complement human work at the task level, our prompt to Llama 3 mentions only replacement. This turned out to be the best option because substitution is a clearer and more discrete concept: a task is either replaceable or not, whereas complementarity can vary in degree and is harder to define consistently. This makes the evaluation easier for Llama 3, especially since we are using its smaller, more accessible version.

l AISE rankings and the manually validated startups-jobs matching dataset (with a balanced number of positive and negative examples) are available at the following GitHub repository: https://github.com/AISE-Data/AISE.

## Preprints

This manuscript was posted on a preprint: arXiv preprint arXiv:2412.04924.
