# Supplementary material for: Follow the money: A startup-based measure of AI exposure across occupations, industries, and regions
Source: PNAS Nexus. 2026 Jun 23;5(6):pgag185. doi: 10.1093/pnasnexus/pgag185 (PMC13288440; doi:10.1093/pnasnexus/pgag185)
Supplement: pgag185_Supplementary_Data [file pgag185_supplementary_data.pdf]

Supplementary Information for *"Follow the Money: A Startup-Based Measure of AI Exposure Across Occupations, Industries, and Regions"*

Enrico Maria Fenoaltea[1,2*], Dario Mazzilli[1], Aurelio Patelli[1], Angelica Sbardella[1*], Andrea Tacchella[1], Andrea Zaccaria[1,3], Marco Trombetti[4], Luciano Pietronero[1]

[1]Centro Ricerche Enrico Fermi (CREF), Via Panisperna 89 A – 00184 Roma
[2]Universitat de Barcelona Institute of Complex Systems (UBICS), Universitat de Barcelona, 08028 Barcelona, Spain
[3]ISC-CNR, Via dei Taurini 19, 00185, Roma, Italy
[4]Translated srl, Via Nepal, 29 00144 Rome, Italy
[*]Corresponding authors: Enrico Maria Fenoaltea (email: enricofenoaltea@hotmail.it); Angelica Sbardella (email: angelica.sbardella@cref.it)

Contents

1 Most and least exposed jobs according to the Occupational AISE index 2

2 Exposure indices vs education and training 5

3 Exposure indices vs number of *crucial* skills 6

4 Skill importance in the AISE-AIOE space 8

5 AISE vs AI Complementarity index 9

6 Test with different input, prompt, and model 11

7 Geographical and sectoral AI exposure 15

8 Robotic Startup Exposure 17

9 AISE with EU startups data 19

10 Yearly Distribution of AI Startups 22

11 YC Startups vs Sectors 25

12 AI Startup Exposure computed with Detailed Work Activities 27

1 Most and least exposed jobs according to the Occupational AISE index

In the tables below, we show the most and least exposed jobs to startups AI applications, according to our AISE index.

As emphasized in the main text, jobs most exposed to AI startup applications share a common set of tasks (see Table 1). These roles predominantly involve processing and analysing information, often in structured formats, along with planning and organization. They typically do not demand significant social interaction but frequently require computer and programming skills. Consequently, these tasks often involve working with a monitor as an essential tool for mediation.

In contrast, the least exposed jobs (see Table 2) and their associated tasks fall into more diverse categories, such as medical specialists, technical workers, and religious officials. These positions are not the primary targets of startups for various reasons, many of which are quantitatively analysed in the main text. For instance, these roles require complex manual skills, frequent social interaction and emotional intelligence, and often involve dealing with ethical and moral issues or with high-risk situations where even a single error can have serious consequences

The case of the airline pilot is particularly noteworthy. One might assume that this role is highly exposed to AI, given that modern airplanes already incorporate high levels of automation for all phases of flight. However, it is crucial to remember that AISE derives its insights from the most recent AI-based startups. Consequently, it is not necessarily evident that new startups would aim to automate tasks that have long been managed by existing technologies.

Supplementary Table 1: **Most exposed jobs according to the AISE index.**

**Job title**	**Job description (O*NET)**
Office Clerks, General	Perform duties too varied and diverse to be classified in any specific office clerical occupation, requiring knowledge of office systems and procedures. Clerical duties may be assigned in accordance with the office procedures of individual establishments and may include a combination of answering telephones, bookkeeping, typing or word processing, office machine operation, and filing.
Data scientist	Develop and implement a set of techniques or analytics applications to transform raw data into meaningful information using data-oriented programming languages and visualization software. Apply data mining, data modeling, natural language processing, and machine learning to extract and analyze information from large structured and unstructured datasets. Visualize, interpret, and report data findings. May create dynamic data reports.
Interviewers, Except Eligibility and Loan	Interview persons by telephone, mail, in person, or by other means for the purpose of completing forms, applications, or questionnaires. Ask specific questions, record answers, and assist persons with completing form. May sort, classify, and file forms.
Computer and Information Systems Managers	Plan, direct, or coordinate activities in such fields as electronic data processing, information systems, systems analysis, and computer programming.
Executive Secretaries and Executive Administrative Assistants	Provide high-level administrative support by conducting research, preparing statistical reports, and handling information requests, as well as performing routine administrative functions such as preparing correspondence, receiving visitors, arranging conference calls, and scheduling meetings. May also train and supervise lower-level clerical staff.
Market Research Analysts and Marketing Specialists	Research conditions in local, regional, national, or online markets. Gather information to determine potential sales of a product or service, or plan a marketing or advertising campaign. May gather information on competitors, prices, sales, and methods of marketing and distribution. May employ search marketing tactics, analyze web metrics, and develop recommendations to increase search engine ranking and visibility to target markets.

Supplementary Table 2: **Least exposed jobs according to the AISE index.**

**Job title**	**Job description (O*NET)**
Clergy	Conduct religious worship and perform other spiritual functions associated with beliefs and practices of religious faith or denomination. Provide spiritual and moral guidance and assistance to members.
Acupuncturist	Diagnose, treat, and prevent disorders by stimulating specific acupuncture points within the body using acupuncture needles. May also use cups, nutritional supplements, therapeutic massage, acupressure, and other alternative health therapies.
Pediatric Surgeons	Diagnose and perform surgery to treat fetal abnormalities and birth defects, diseases, and injuries in fetuses, premature and newborn infants, children, and adolescents. Includes all pediatric surgical specialties and subspecialties.
Terrazzo Workers and Finishers	Apply a mixture of cement, sand, pigment, or marble chips to floors, stairways, and cabinet fixtures to fashion durable and decorative surfaces.
Nurse Anesthetists	Administer anesthesia, monitor patient's vital signs, and oversee patient recovery from anesthesia. May assist anesthesiologists, surgeons, other physicians, or dentists. Must be registered nurses who have specialized graduate education.
Airline Pilots, Copilots, and Flight Engineers	Pilot and navigate the flight of fixed-wing aircraft, usually on scheduled air carrier routes, for the transport of passengers and cargo. Requires Federal Air Transport certificate and rating for specific aircraft type used. Includes regional, national, and international airline pilots and flight instructors of airline pilots.

2 Exposure indices vs education and training

In the main text, we illustrated how the AISE index varies across job zones within fixed intervals of AIOE. Our findings indicate that, for a given AIOE interval, jobs that demand higher levels of education and training are less likely to be targeted by AI-based startups. The figure below displays the overall relationship between job zones and the AISE, independent of AIOE. For comparison, we also present the general relationship between job zones and AIOE.

The figure shows that both indices increase from job zone 1 to job zone 4. The prevalence of manual labour in the lower job zones is the main driver of reduced AI exposure. However, only our AISE index exhibits a notable decline between job zone 4 and job zone 5. This difference arises because, unlike the AIOE, which is an ability-based index that considers jobs requiring more cognitive skills as more exposed, our AISE index reflects the genuine interest of the startup market in AI applications. As highlighted in the main text, jobs demanding the highest levels of education and training often entail significant ethical and social considerations and involve higher-risk scenarios, making the integration of AI less straightforward compared to most jobs in job zone 4.

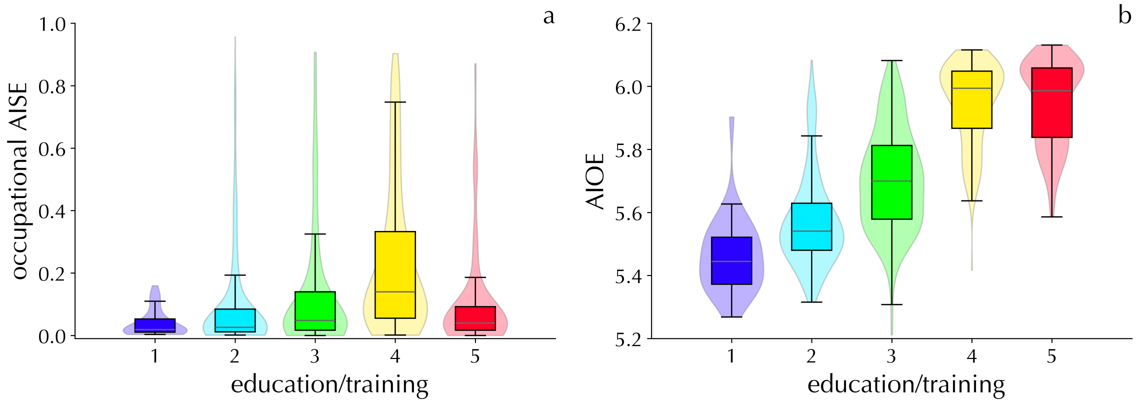

Supplementary Figure 1: **AISE and AIOE vs Job zone.** The width of the violin is proportional to density of jobs within the corresponding interval of exposure values and the horizontal line represents the average.

3 Exposure indices vs number of *crucial* skills

The two figures below illustrate how the average AIOE and AISE for jobs vary with the number of skills required that have an O*NET importance score greater than 4. Consistent with the literature suggesting that high-skilled jobs are the most exposed, we observe in Fig.2 that the AIOE increases with the number of skills required that have an importance score above 4. However, for jobs within a fixed AIOE range, the AISE index decreases as the number of required skills with an importance score greater than 4 increases. In Fig.3 we further unpack this observation, by looking at how AISE behaves in professions with different ranges of crucial skills at varying levels of theoretical exposure as measured by AIOE (divided in three ranges containing the same number of professions). The decreasing trend is particularly evident for professions with high theoretical exposure (panel C), strongly suggesting that the combination of many crucial skills is an important discouraging factor in attempting automation.

This reflects that, given a fixed theoretical automation potential (AIOE), there is a preference—especially from Y Combinator startups—to invest in AI applications targeting the automation of professions that require a less sophisticated and rich mixture of skills.

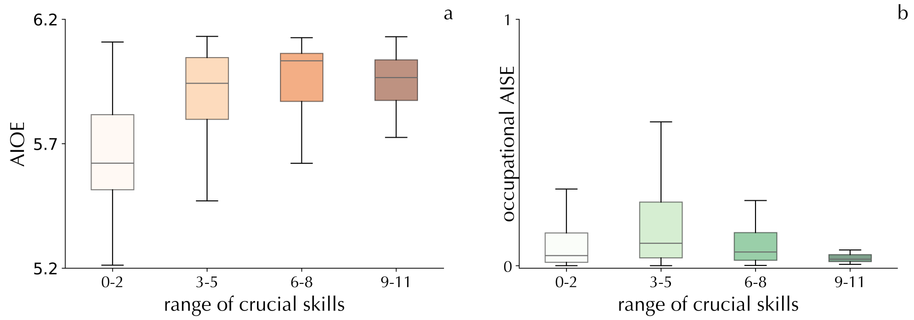

Supplementary Figure 2: **AIOE vs number of skills with importance larger than 4 (crucial skills).**

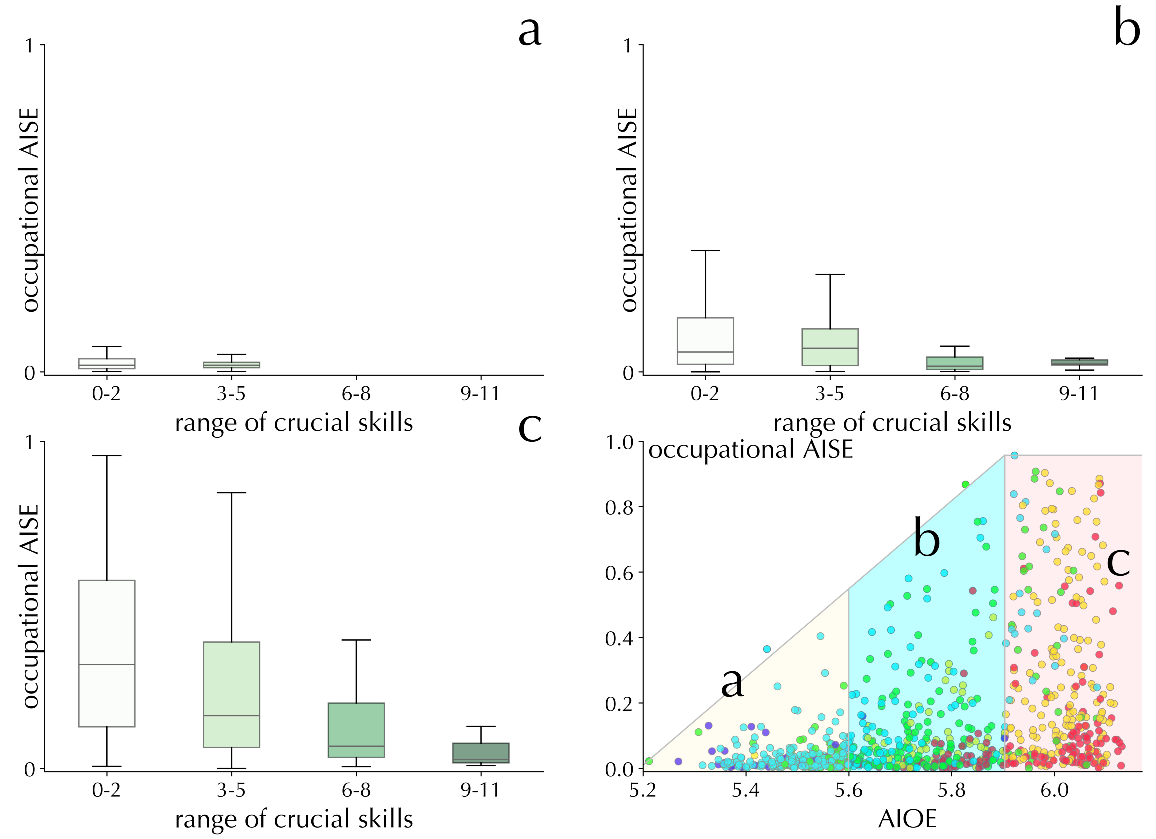

Supplementary Figure 3: **AISE vs number of skills with importance larger than 4 (crucial skills) for different fixed ranges of AIOE values.** The three ranges of AIOE values -*a*, *b* and *c*- are represented in the fourth panel. Each of the three sections contains the same number of professions.

4 Skill importance in the AISE-AIOE space

In the main text, we analyzed the frequency of required skills with an O*NET importance score exceeding 4 for jobs in regions characterized by high AIOE and high AISE, as well as high AIOE and low AISE. Fig. 4 displays a similar bar plot, but with the importance threshold for required skills lowered to 3. The findings are consistent with those presented in the main text. Jobs in the lower-right region of the AIOE-AISE space show a significantly higher likelihood of requiring skills with an importance score above 3. Additionally, skills related to social and human domains, such as *social perceptiveness* and *instructing*, are more frequently observed in this area.

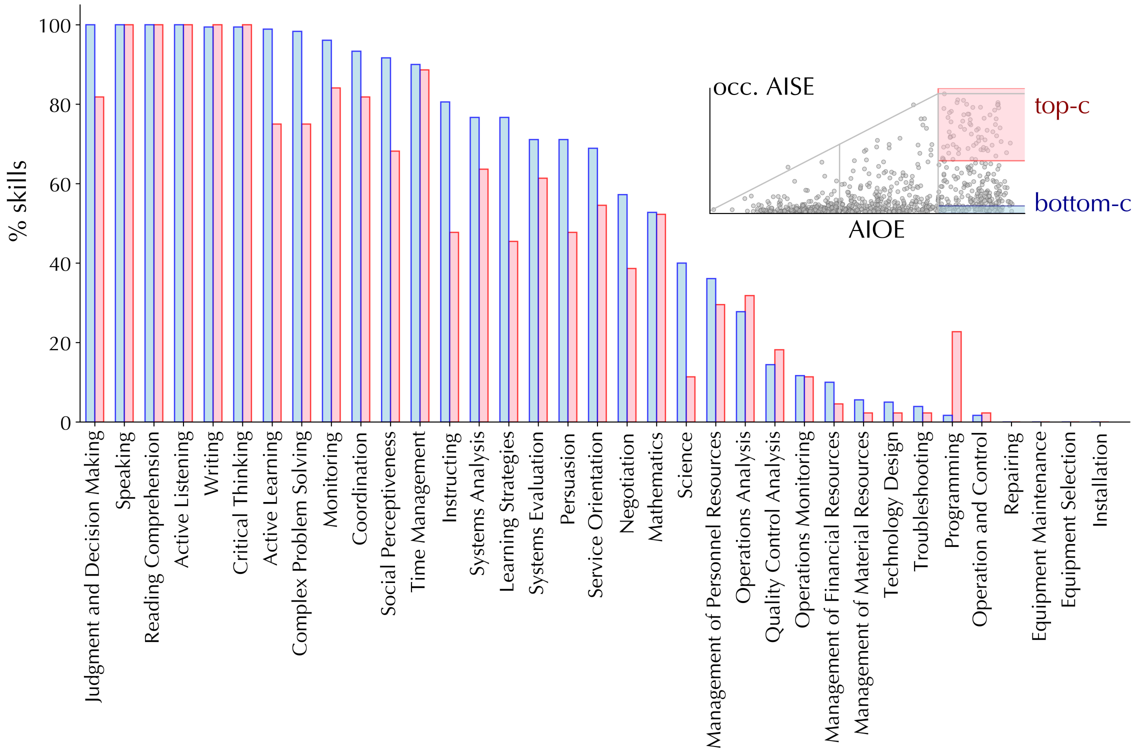

Supplementary Figure 4: **Frequency of skills with importance larger than 3 for two different region of the AISE-AIOE space.** Blue bars describe the jobs in the top right part of the AISE-AIOE space; red bars describe the jobs in the bottom right part of the AISE-AIOE space. For each skill, the smaller bar is over the higher bar.

5 AISE vs AI Complementarity index

As mentioned in the main text, both AIOE and AISE evaluate the exposure of occupations to AI without distinguishing between complementary and substitutive effects. In the case of complementarity, AI acts as an enhancement to human work; conversely, in the case of substitution, AI could render the worker's presence unnecessary. An approach proposed to account for these two effects was introduced in Pizzinelli et al. (2023).

In that work, the authors define a potential complementarity metric θ, still based on the O*NET dataset but exploiting the *work contexts* together with Job Zones. The author propose a classification of work contexts that are more or less prone to acceptable delegation to AI and compute their complementarity metric as a weighted average on that basis.

In Fig. 5, occupations are binned into hexagonal cells in the AISE–AIOE space, and each cell is coloured according to the average complementarity index of its occupants. As shown in the figure, for a fixed level of AIOE, occupations with lower AISE tend to have a higher complementarity index. Of course, we acknowledge that the complementarity index introduced here is not necessarily the definitive or best measure of complementarity, as the very notion of complementarity is not sharply defined and could admit different interpretations (e.g., whether complementarity implies augmentation or only partial substitution). Nevertheless, the figure suggests that even though AIOE and AISE alone do not reveal the type of AI exposure, their combination can provide useful insights. In particular, the figure suggests that occupations with high AIOE (high technological feasibility) and low AISE (little current targeting by startups, for the reasons discussed in the main text) are those where AI has the greatest potential for complementary application.

This analysis can serve as a first step toward a more systematic exploration of the potential effects of AI on occupations from the AISE perspective.

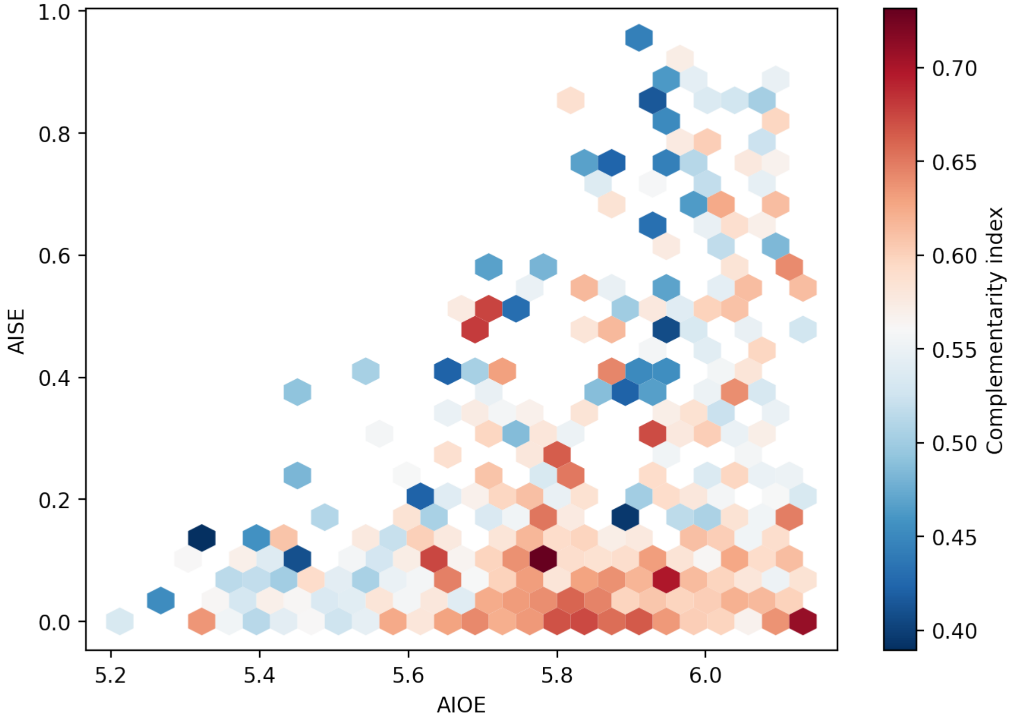

Supplementary Figure 5: **AISE vs AIOE colored with complementarity index.** Occupations are binned into hexagonal cells in the AISE–AIOE space, and each cell is coloured according to the average complementarity index of its occupants.

6 Test with different input, prompt, and model

As mentioned in the main text, to evaluate the robustness of our methodology and findings, we repeated the experiment under two different conditions. In the first scenario, we feed Llama3 with the same prompt as before but replaced the detailed descriptions of the startups with shorter ones provided by Y Combinator.

For example, while the detailed description for the startup *Studdy* is :

"At Studdy, our mission is to unlock the full potential of the next generation by providing a personalized AI tutor for every student. Studdy Buddy is a multilingual tutor that uses AI, as well as advanced speech, text, and image recognition technology to supercharge students' ability to learn new subjects. We believe that making self-learning as easy as possible for as many students as possible (no matter their cultural, social, or educational background) is the key to unlocking the full potential of students around the world. We're a passionate team of AI experts, educators, and builders - if you also have a passion for transforming education we'd love to hear from you. Shoot us a message at team@studdy.ai!".

Instead, the short description is: "An AI math tutor for every student".

While the detailed descriptions are more informative, they often include extraneous details about the founders or funding. On the other hand, the shorter descriptions are more focused on the product being developed, minimizing the likelihood of Llama3 being influenced by irrelevant details. Nevertheless, as shown in Fig. 6, the results with the shorter descriptions closely align with those obtained from the detailed descriptions. In particular, the pearson correlation coefficient is 0.91, while the Kendall coefficient, a metric that measures the ordinal association between two variables, is 0.74.

In the second scenario, we replace the prompt used for the results in the main text:

{*"role": "system", "content": "You are an AI specialist."*}
{*"role": "user", "content": "Given the following startup description: " + startup[j] + "and given the following job description: "+ job[i] + "can the product or service developed by the startup help directly replace humans in performing some of the described job's tasks? Use only the information provided by the two descriptions. Reply only yes or no."*}

with the following revised prompt:

{*"role": "system", "content": "You are an AI specialist."*}
{*"role": "user", "content": "Given the following startup description: " + startup[j] + "and given the following job description: "+ job[i] + "Is the product or service developed by the startup designed to directly replace humans to perform some of the described job's tasks? Use only the information provided in the two descriptions. Reply only with yes or no."*}

This revised prompt takes a more direct approach by explicitly asking whether the startup's product or service is designed to replace human labour for specific tasks mentioned in the job description. In contrast, the previous prompt gave Llama 3 more interpretive freedom to assess the relationship between the startup's AI product/service and the job's tasks. Again, despite these differences in framing, the exposure rankings generated by the two prompts remain highly consistent, as shown

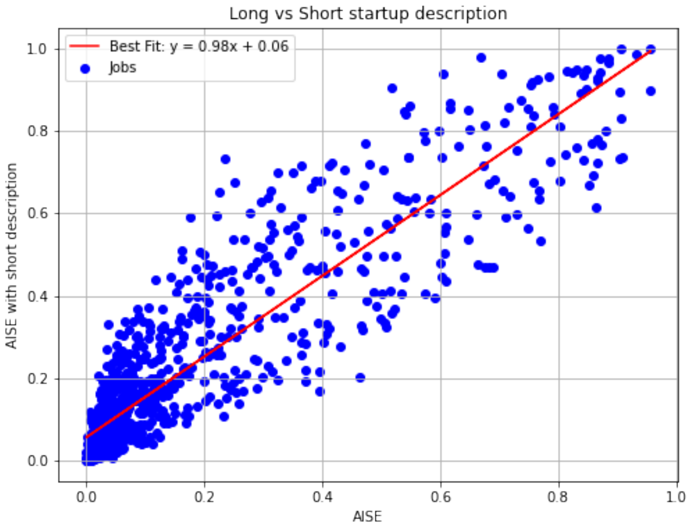

Supplementary Figure 6: **Scatter plot comparing the standar occupational AISE with the AISE constructed with the short startup description provided by Y Combinator. The Pearson's correlation coefficient is 0.91.**.

in Fig.7. In particular the Pearson correlation coefficient is 0.97, while the Kendall coefficient is 0.85.

As an additional robustness test, we computed the AI exposure for a subset of occupations using a more powerful LLM: GPT-4o. GPT-4o is OpenAI's flagship model, released in May 2024. It combines advanced reasoning capabilities with high-speed performance and is estimated to contain over one trillion parameters. Unlike Llama 3, GPT-4o is not open-source and is only accessible via a paid API. Due to these access constraints, we ran this analysis on a limited set of occupations rather than the full dataset.

In general, given the same prompt described in the Methods section of the main paper, the results obtained using GPT-4o are qualitatively consistent with those from Llama 3. In the table below, we compare the AISE values produced by the two models for five occupations that summarize the main takeaway of our paper. Specifically, *Executive Secretaries and Executive Administrative Assistants* and *Data Scientists* are occupations considered highly exposed to AI by both AISE and standard AI exposure indices in the literature. In contrast, *Judges, Magistrate Judges, and Magistrates*, *Anthropology and Archeology Teachers*, and *Family Medicine Physicians* receive high scores under traditional indices such as AIOE but low AISE values (as discussed in detail in the main paper). As shown in the table, while the absolute values differ, the AISE scores computed with GPT-4o and Llama 3 reveal the same relative ranking and the same message: many startups target jobs involving information processing and planning skills, while high-stakes occupations with strong ethical or responsibility-related content are much less targeted.

There are, however, a few important remarks to make. First, given the same prompt, GPT-4o is more conservative in returning "yes" when asked whether a startup's product or service

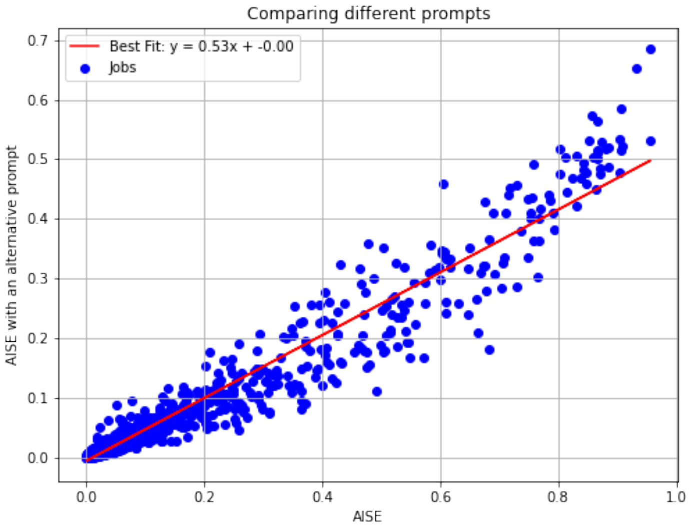

Supplementary Figure 7: **Scatter plot comparing the standar occupational AISE with the AISE constructed by feeding Llama3 with a different prompt. The Pearson's correlation coefficient is 0.97.**.

can substitute some tasks described in a given occupation. Accordingly, AISE scores computed with GPT-4o are generally lower than those computed with Llama 3. A proper upgrading of the analysis to a different and larger language model would thus entail a calibration of the scores or experimentation with the prompt structure. Moreover, GPT-4o appears more sensitive to subtle variations in the prompt. For example, consider the question: *"Can the product or service developed by the startup directly replace humans to perform some of the described job's tasks?"* While adding or removing the word "directly" has virtually no impact on Llama 3's response, it has a strong effect on GPT-4o, which becomes significantly more conservative—only answering "yes" when the startup description explicitly mentions core tasks in the job description. This is consistent with expectations: Llama 3 8B is a relatively small model and thus less capable of interpreting prompt subtleties, whereas GPT-4o is a large-scale reasoning model able to handle such nuances.

Finally, we stress that by relying on Llama 3, we knowingly trade off some precision and introduce a bit more noise. However, we do so to ensure accessibility and full reproducibility, as Llama 3 8B is freely available, open-weight, and can be run locally on modest hardware—making our analysis more transparent and replicable.

**Occupation**	**Llama 3 (8B)**	**GPT-4o**
Data Scientists	0.963	0.522
Executive Secretaries and Executive Administrative Assistants	0.954	0.277
Family Medicine Physicians	0.059	0.070
Anthropology and Archeology Teachers	0.035	0.040
Judges, Magistrate Judges, and Magistrates	0.008	0.001

Supplementary Table 3: Comparison of AISE values calculated with Llama 3 (8B) and GPT-4o for selected occupations.

7 Geographical and sectoral AI exposure

In this section, we show in Figure 8 the average Occupational AISE of US counties. Occupational employment data at the county level is not available while industry-level employment information is, to obtain Geographic AISE at the county level we thus project the national level occupational composition at the county level by linking occupations to industries. While this results in a noisier information and counties that have a small population tend to have more uncertainty on the exposure measure, we project our indicator at the county-level to compare it with Felten et al. (2018)'s findings. As can be observed in the map, the geographical distribution of colour is consistent with that observed in the map in Figure 4 of the main text.

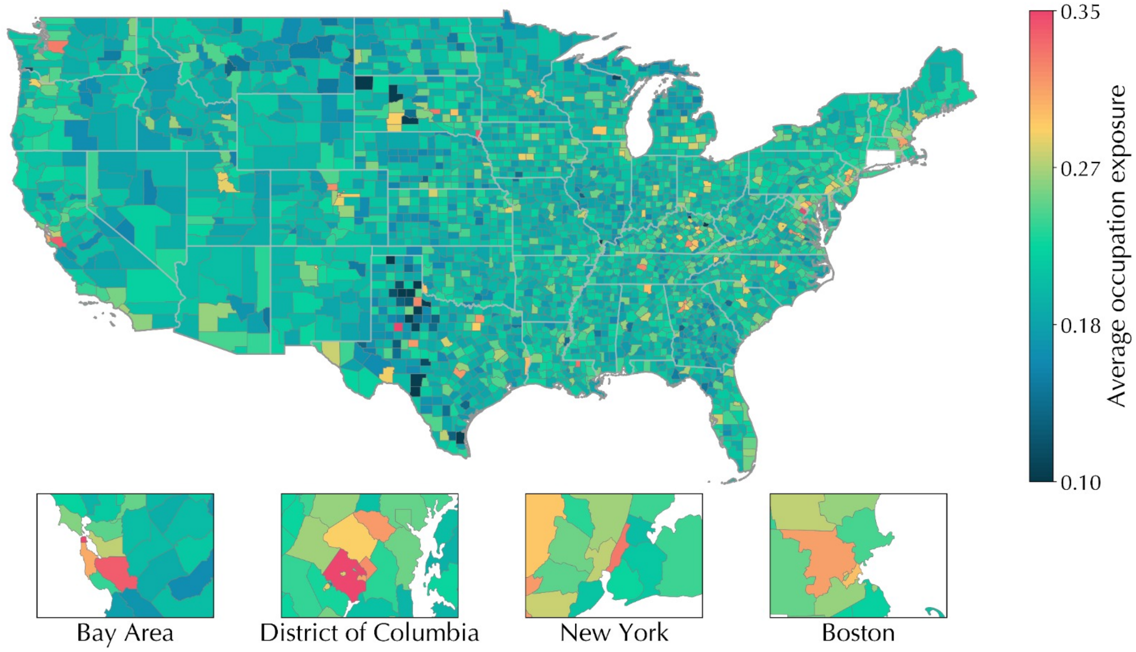

Supplementary Figure 8: **County-level Geographic AISE**. The colour-coding of the figure indicates the Geographic AISE of US counties. At the bottom panels we zoom-in the four most exposed counties.

In Figures 9 and 10 we show the relationship between our Geographical and Sectoral AISE measures and Felten et al. (2021)'s AIGE (AI Geographic Exposure) and AIIE (AI Industry Exposure) indices, respectively. Both scatter plots show a clear positive association between our AISE-based measures and Felten et al.'s indices: counties with higher AIGE tend to have higher geographical AISE, and industries with higher AIIE tend to have higher sectoral AISE. At the same time, the dispersion around the upward trend mirror the differences found when analysing occupational exposure rankings.

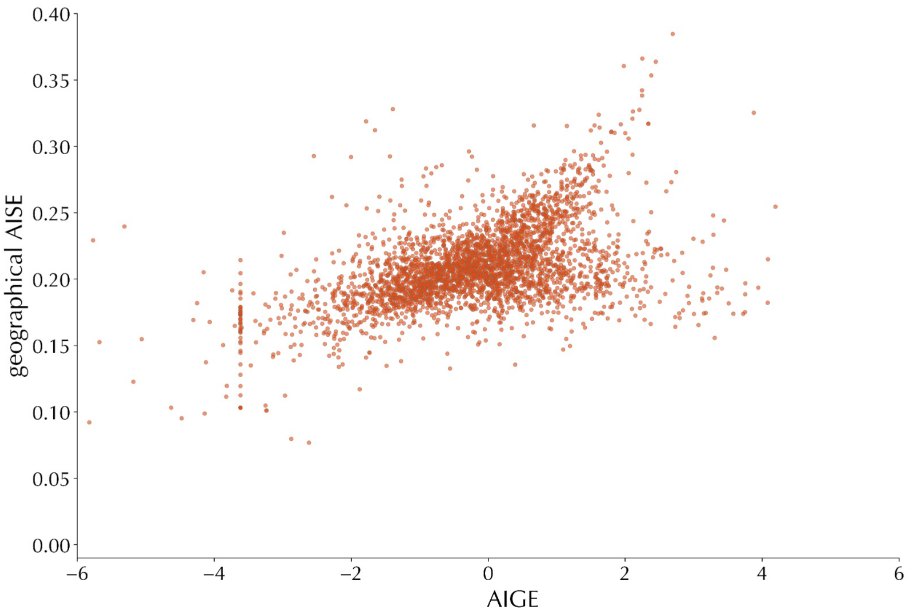

Supplementary Figure 9: **Geographical AISE vs AIGE**. Each point represents a US county.

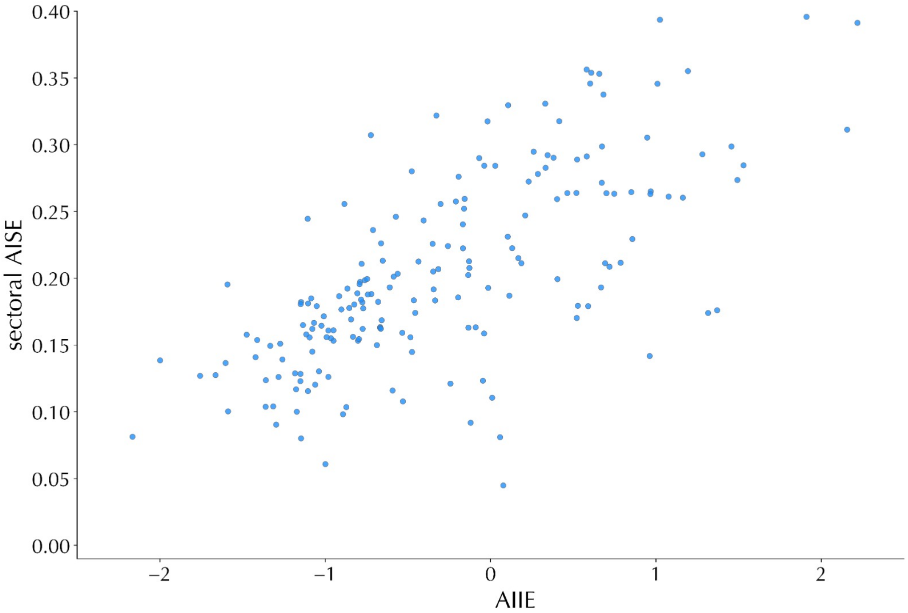

Supplementary Figure 10: **Sectoral AISE vs AIIE**. Each point represents an industry.

8 Robotic Startup Exposure

In this section, we demonstrate how the methodology used to compute the AISE can be generalized to study the forward-looking impact of robotics and its integration with AI on the labour market. While there is a long-standing body of literature on the impact of robotics and automation (Acemoglu and Autor, 2011; Acemoglu and Restrepo, 2020; Graetz and Michaels, 2018), interest in the impact of AI integration with robotics is more recent, with many believing that it will have an even more radical impact than AI alone (Soori et al., 2023; Barbieri et al., 2020). As we show below, our framework allows us to observe directly the impact of the joint combination of AI and robotics, as most of the Y Combinator startups with a robotic-related tag are integrating AI software with robot's hardware.

Adopting the same strategy we used to build AISE, we feed Llama3 the SOC occupations' textual descriptions and the descriptions of all the AI startups that are also associated with a robotics-related tag on Y Combinator. Therefore, for each occupation, we define the Robotic Startup Exposure (RSE) index as the normalized number of startups developing robotic applications identified by the LLM as substitutes for one or more of the essential tasks described in the O*NET short descriptions. It is important to notice the exploratory nature of this analysis, in fact only about 100 startups with a robotics-related tag are present in the dataset, compared to approximately 1,000 AI-tagged startups.

Figure 11 details our findings. The left panel for each occupation shows a AISE versus RSE scatter plot, colour-coded according to job zones. Interestingly and unsurprisingly, when robotics is considered, the pattern of exposure changes. In fact, several occupations with low AISE scores actually display a high RSE. As illustrated in the right panel, this is particularly evident for professions in lower job zones, which require more manual abilities or skills (e.g., *Control Movement Abilities*). Instead, professions in job zone 5, thus requiring high levels of educations, present low values for both AISE and RSE.

A peculiar finding is that jobs with high AISE also have high RSE, despite these jobs (such as *Office Clerk, General*) do not require any physical skills. This is because, as already mentioned, in the Y Combinator dataset, most startups with a robotics-related tag also have an AI-related tag[1]. Therefore, we are observing startups developing AI products integrated into hardware, and Llama 3 considers a job exposed to these startups even if it is exposed only to the AI software component of the products they develop.

Overall, these preliminary results suggest that the joint action of AI and robotics will be pervasive across all occupations, warranting further in-depth studies. Such potential developments, however, would require broader and more complete data sources to cover a more significant number of startups, as well as further methodological developments that allow for a disentanglement the AI and Robotics effects on impact.

[1] This also explains why there are only about a hundred startups with robotics-related tags. Indeed, the technology that combines AI and robotics is still in its early stages, and there is still significant uncertainty regarding its reliability and adoption (Eloundou et al., 2024).

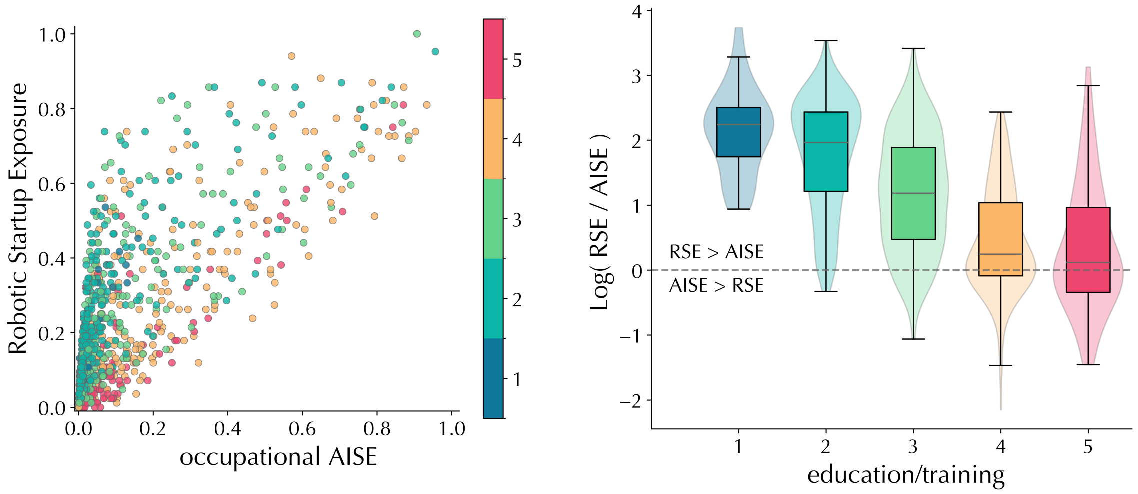

Supplementary Figure 11: **Comparison of the Robotic Startup Exposure with AISE and the educational levels**. The left panel shows the scatter plot of the Robotic Startup Exposure with the occupational AISE. Each dot represents an O*NET job and the color code indicates the educational/training level of the job accessible from the O*NET database. The right panel shows a violin plot of the relative importance of the two exposure measures subdivided by education/training level.

9 AISE with EU startups data

In this section we repeat the analysis using a different data set as a robustness check. In figure 12, we show the relationship between the AISE computed using Y Combinator startups and the AISE based on the EU-Startups dataset. EU-Startups is a leading online publication focused on startups across Europe, founded in 2010. The website covers startups from all EU member states as well as the United Kingdom, Switzerland, and other European countries. Its startup directory features over 30,000 companies operating in the European tech ecosystem. From this dataset, we selected all startups tagged with at least one of the 68 AI-related labels (not listed here for brevity). To ensure consistency with the Y Combinator sample and to focus on credible, well-funded ventures, we further restricted the selection to those startups reporting a funding amount of at least €500,000. Funding levels in the EU-Startups database are reported in intervals, with the highest category being "above €25 million." For each selected startup, we extracted and concatenated the "business description" and "long business description" fields to use as input for Llama 3. After filtering by AI tags and funding level, we obtained a sample of 830 AI startups, founded between 2005 and 2024 (again, with the majority established in the past ten years).

We observe (Fig.12) that the correlation between the AISE computed with Y-Combinator firms and with EU-Startups is remarkably high, with an R^2 of 0.9 (Pearson correlation is 0.98). Moreover, Figure 13 demonstrates that when plotting the EU-based AISE against the AIOE—as done in Figure 1 in the main text—the same patterns observed with the Y Combinator data clearly emerge. This suggests that all findings presented in the main text remain valid when using the EU Startups data.

As also noted in the main text, we chose not to merge the two datasets because they differ in key dimensions, including funding levels, investor profiles, geographic focus, and structural aspects such as the format and content of the startup descriptions. We opted to report results based on the Y Combinator dataset for both brevity and consistency. Unlike the EU Startups database, where funding comes from diverse sources and varies across ranges, and where the quality of the records can be heterogeneous, all Y Combinator startups are funded under the same funding scheme, after comparable high-quality scrutiny and where information about the company and the received funding is completely certain and timely.

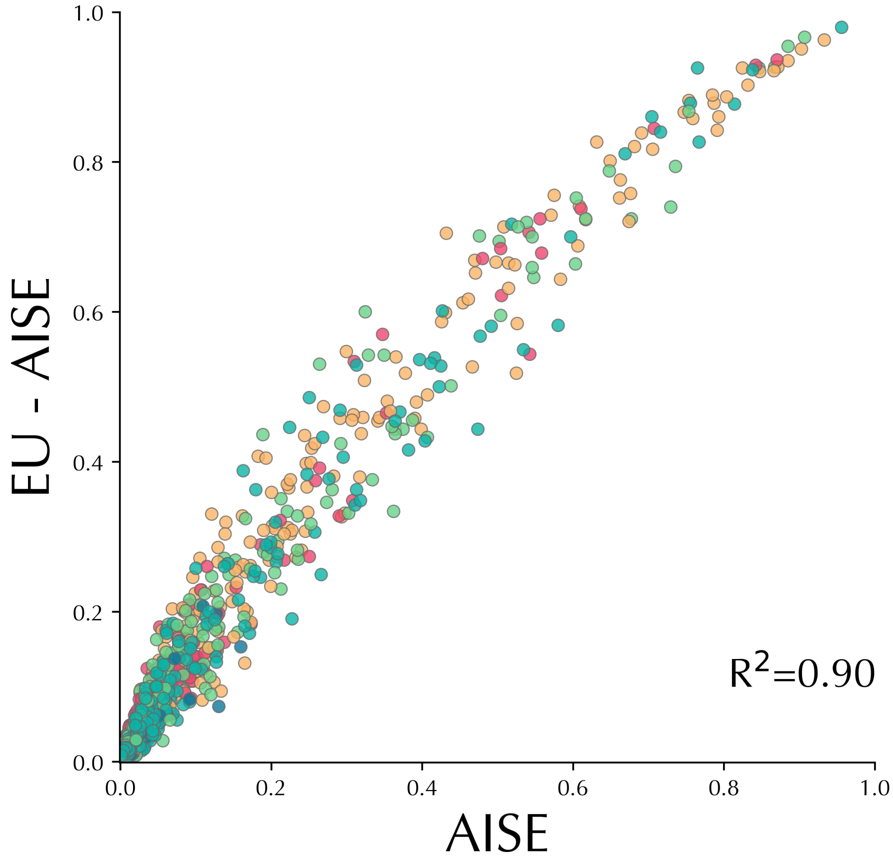

Supplementary Figure 12: **Correlation between the Y Combinator–based AISE and the EU Startups–based AISE.** Each point represents an occupation and the color is the corresponding job zones (see legend in Fig.13)). The strong linear relationship demonstrates the consistency of the AISE across the two independently constructed datasets.

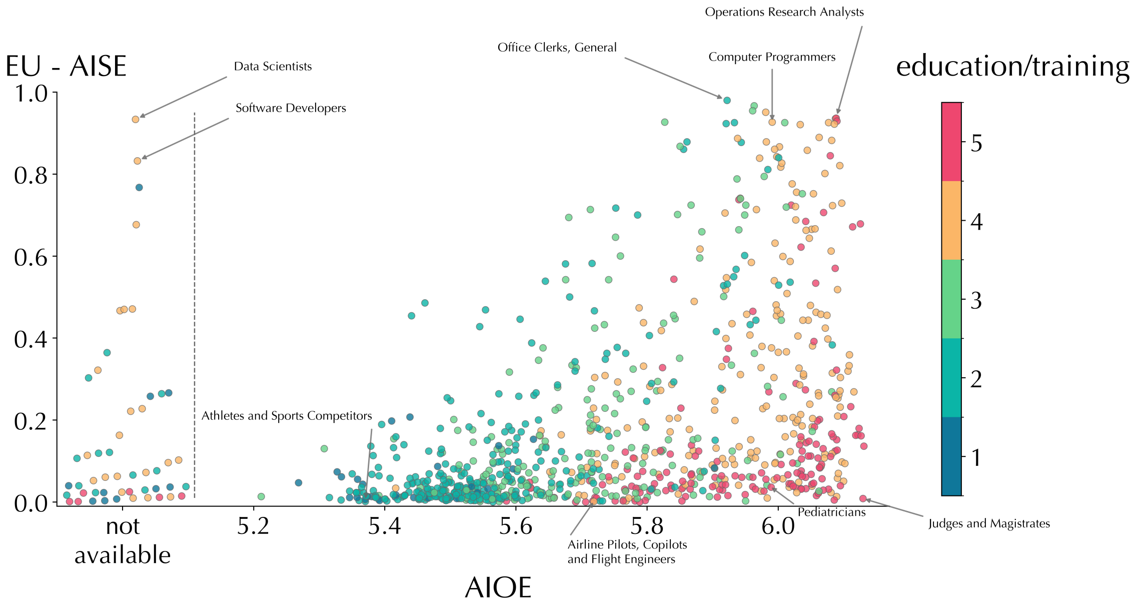

Supplementary Figure 13: **EU Startups–based AISE vs AIOE.** Each point represents an occupation, with colors encoding the corresponding job zone. The pattern that emerges is the same shown in Figure 1 of the main text

10 Yearly Distribution of AI Startups

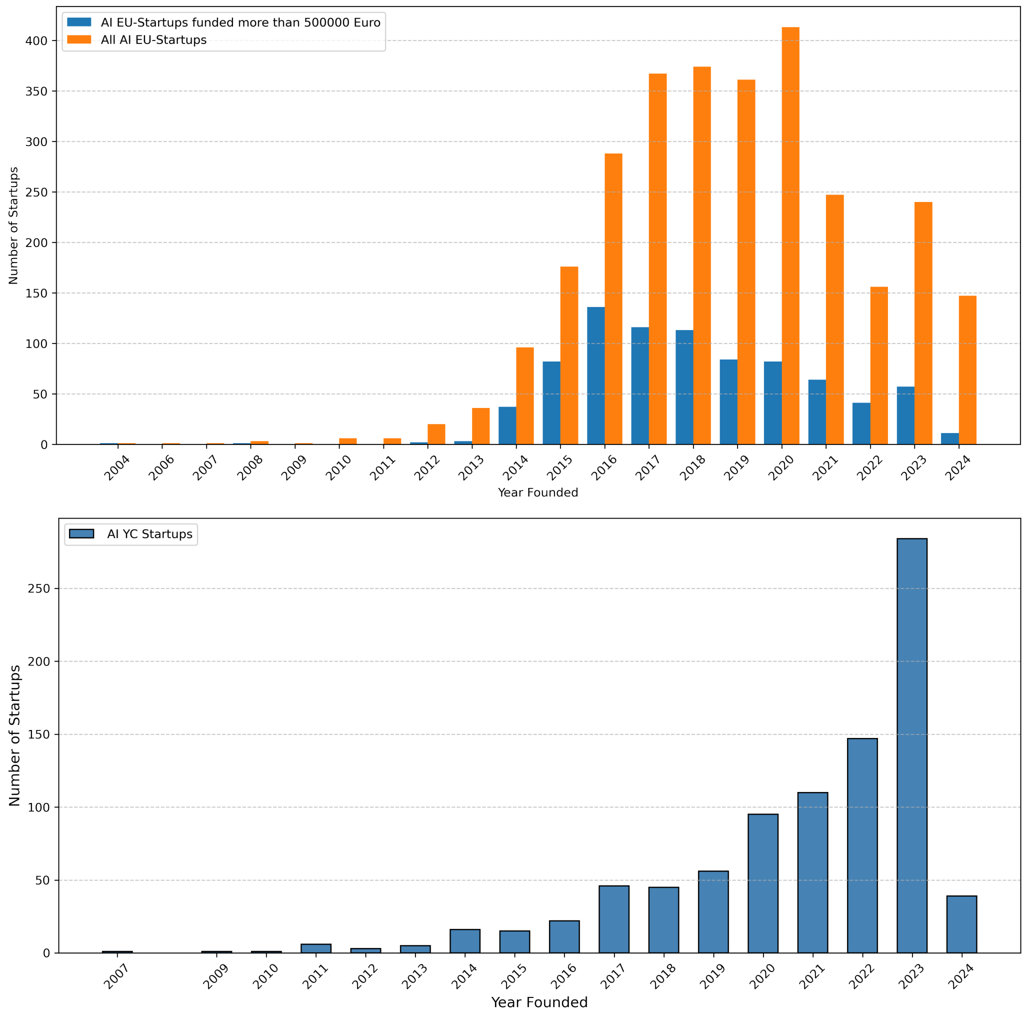

Supplementary Figure 14: **Number of AI startups founded per year.** The two plots show the yearly founding trends for startups in the EU-Startups dataset and Y Combinator dataset, respectively.

In Figure 14, we show the number of AI startups founded each year in both the EU-Startups dataset (top panel) and the Y Combinator dataset (bottom panel). In the EU-Startups plot, the orange bars represent all AI startups, while the blue bars indicate only those that received funding of at least half a million euros. Since all Y Combinator startups receive a fixed investment of 500,000 dollars, no such distinction is needed in the bottom panel. Both plots illustrate that the majority of AI startups were founded in the past ten years. The trend is clearly upward, particularly in the Y Combinator dataset. The apparent decline in the EU-Startups dataset after 2019–2020 is likely

due to a lag between the founding date and the receipt of funding—this is visible in the delayed drop in the blue bars relative to the orange ones—as well as possible delays in data collection by EU-Startups. The presence of this lag is one of the reasons we chose to present only the Y Combinator–based results in the main paper, given the higher consistency and quality of the Y Combinator dataset.

Since most startups in the Y Combinator dataset were founded after 2020, in Fig. 15 (top panel) we show the correlation between the AISE index computed on the full dataset and the same index computed using only startups founded after 2020. The correlation is very high, indicating that our analysis would lead to the same conclusions even if we considered only more recent startups. We nevertheless include the entire time span of the dataset both to maintain an adequate sample size and to ensure robustness when comparing with other datasets. Indeed, restricting the analysis to only the past five years would significantly reduce the number of observations, and it would also make it harder to test robustness against other sources, since, as mentioned above, the EU startups dataset shows a time lag in posting the latest startups.

In Fig. 15 (bottom panel), we also show how the AISE index varies when considering startups founded before 2020 versus those founded after 2020. Interestingly, some jobs had a low AISE before 2020, which increased sharply afterward. While this result should be interpreted with caution because the sample size before 2020 is small, it suggests that future research could explore AISE dynamics over time to predict how professions' exposure to startups evolves. As the Y Combinator dataset continues to grow rapidly, such longitudinal analyses will soon become feasible and may provide new insights into job market dynamics.

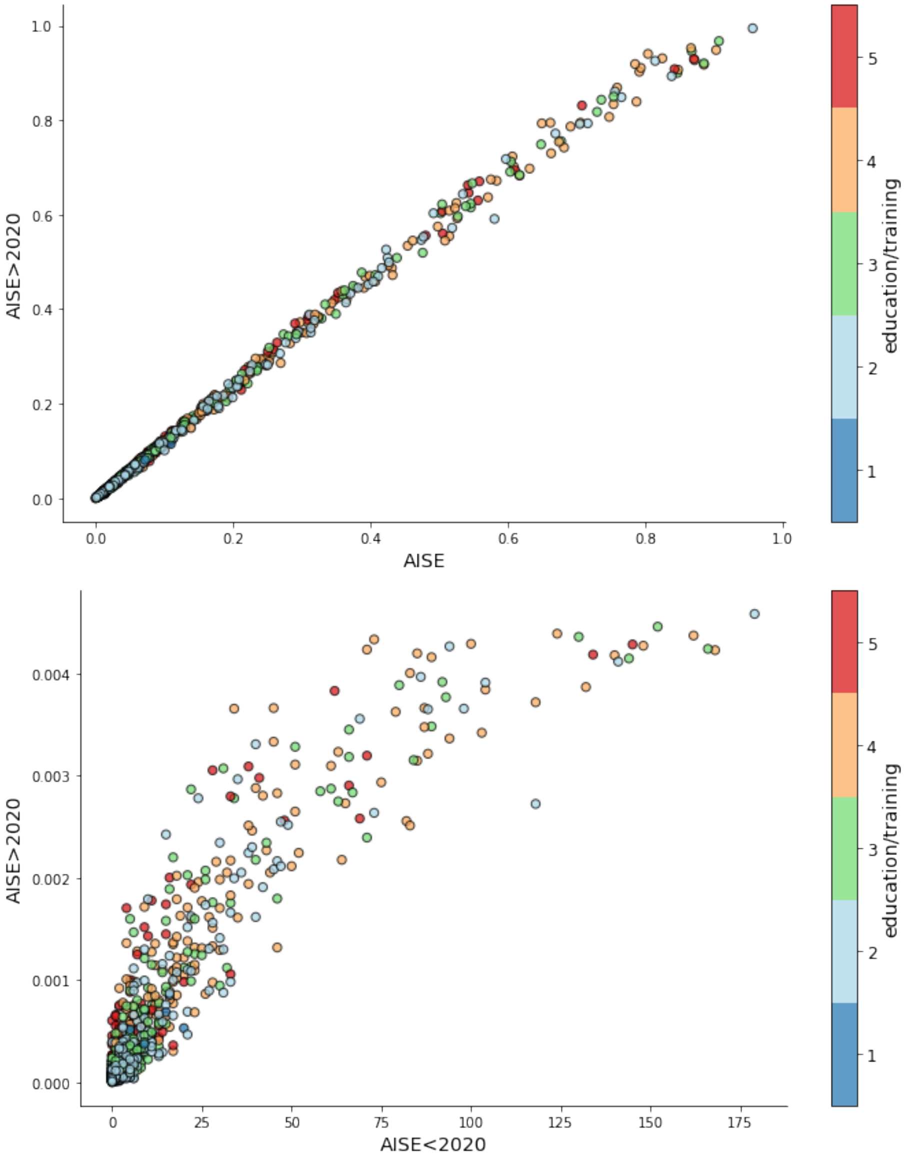

Supplementary Figure 15: **Global AISE vs AISE in specific time intervals.** Top panel: AISE computed using all startups compared with AISE computed using only startups founded after 2020. Bottom panel: AISE computed using only startups founded before 2020 compared with AISE computed using only startups founded after 2020.

11 YC Startups vs Sectors

To show which sectors attract proportionally more investment in AI startups compared with the overall startup landscape, Fig. 16 reports the share of all YC startups labeled with a given industry tag together with the share of AI startups with the same tag. In the YC database, each startup is assigned both an industry tag and a sub-industry tag (different from the tags used to identify AI startups). The tags shown in the figure correspond to the sub-industry tags. These are broad internal labels used by YC to help users filter and compare startups. They are not official NAICS codes, although the taxonomy can be roughly mapped to standard industry categories. The EU startup dataset does not provide a comparable industry classification, so the analysis shown in Fig. 16 is performed only on the YC-funded startups.

The figure shows that, while there is general alignment, i.e., sectors with the most AI startups are often those that attract the most investment overall (they host many startups regardless of whether they are AI or not), some industries behave differently. For example, *Education* is among the most heavily invested sectors overall but has relatively few AI startups. The same pattern holds for industries such as *Social, Food & Beverage*, and *Home & Personal.* Conversely, some industries attract a higher share of AI startups compared with their overall startup presence, such as *Analytics, Sales*, and *Operations.* Finally, AI startups are generally less evenly distributed across sectors compared with the overall startup population. These findings support the consideration that the signals that we observe are not simply driven by the peculiar sectoral distribution of startup investment, as AI-specific startups tend to concentrate differently across sectors, following logics that are specific for this technology.

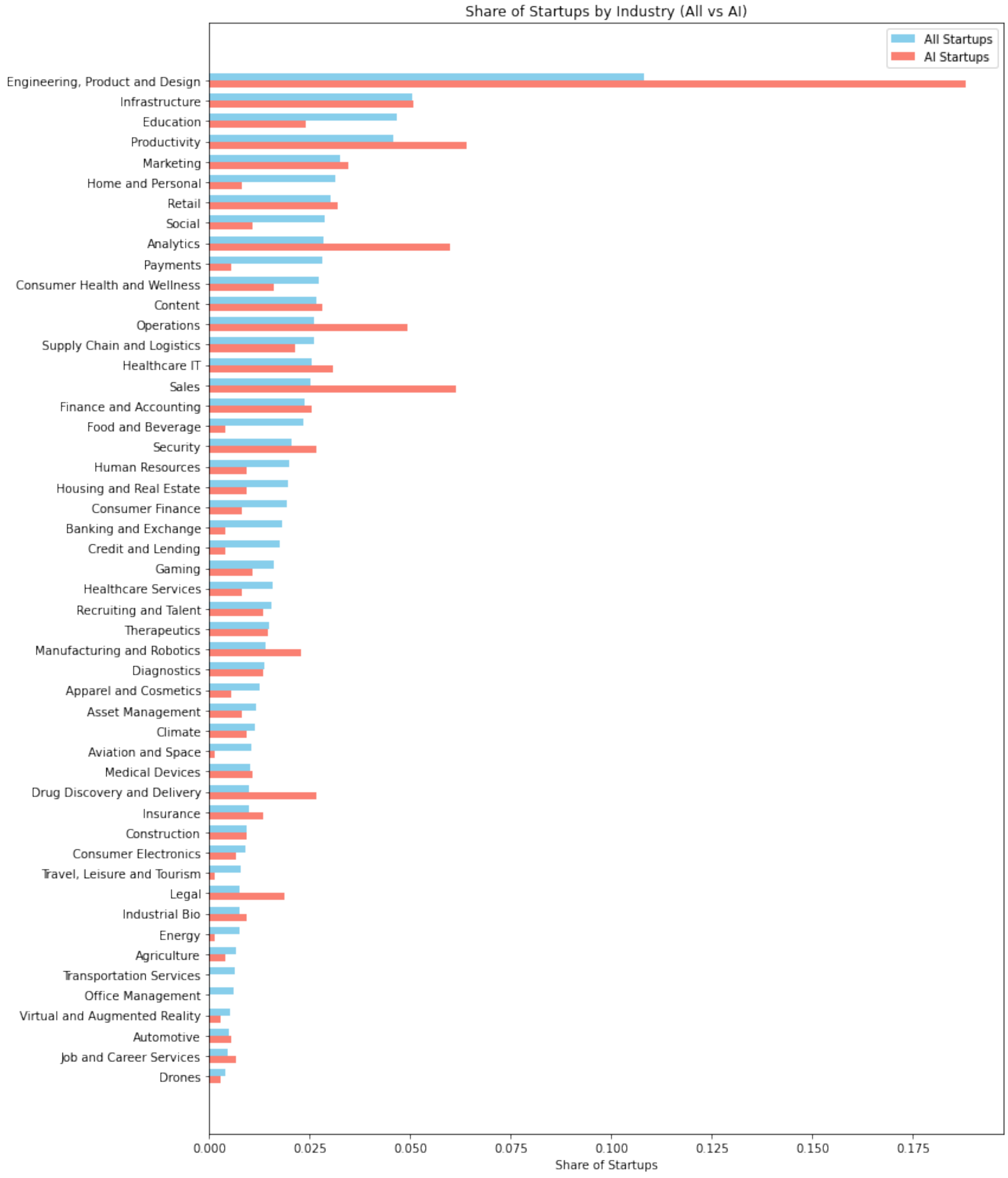

Supplementary Figure 16: **Share of YC startups by YC Industries. Blue bars show the distribution for all YC startups, orange bars show the distribution only for startups with an AI tag.**

12 AI Startup Exposure computed with Detailed Work Activities

Our AISE index was unconventionally constructed using the *short descriptions* of occupations provided by O*NET, rather than the more commonly used job *tasks* associated with each occupation (also provided by O*NET). As explained in the main text, we chose to use O*NET's short occupation descriptions because they better capture the core meaning and context of each job, compared to the list of associated tasks. Indeed, the goal of our methodology is to investigate which AI startups *interact with* which occupations, rather than pinpointing which specific tasks are automated. Our main interest is to capture which areas of the labor market are being targeted by the AI startup ecosystem. This broader perspective is also reflected in the prompt we use (see the Methods section), which gives LLaMA 3 some flexibility in interpretation.

Moreover, relying on tasks can introduce noise, as most occupations include tasks that are easily automatable by AI, but which do not capture what makes the occupation intellectually or socially meaningful. For example, a researcher may perform automatable tasks such as writing reports or analyzing data, yet the core of research work lies in generating new ideas, posing original questions, and contributing to knowledge.

On the other hand, using tasks has the potential advantage of offering a more precise and measurable definition of an occupation's exposure. For instance, one could quantify exposure as the percentage of an occupation's tasks that are highly targeted by AI startups.

For this reason, in this section we perform an additional robustness check by constructing our AISE index using O*NET's *Detailed Work Activities* (DWAs) instead of the short descriptions. DWAs are generalized, transferable job tasks. They are designed to be standardized and decontextualized so they can be reused across occupations and support cross-occupational comparisons. In other words, DWAs are an aggregated version of O*NET tasks, which are more job-specific. We opt to use DWAs instead of tasks primarily for computational efficiency: while tasks in O*NET number over 19000, there are just over 2000 DWAs, meaning that the number of startup–task associations to be evaluated by LLaMA 3 is significantly reduced.

Specifically, mirroring the approach we used with job descriptions, for each DWA we iterate over all AI-tagged startups from Y Combinator and feed the following prompt to LLaMA 3:

{"role": "system", "content": "You are an AI specialist."}
{"role": "user", "content": f"""Startup description: {startup}
Task Description: {DWA}
Based only on the information above, can the startup's product or service directly replace a human in performing the described task?
Answer with only one word: Yes or No"""}

The AI exposure of each DWA is then calculated as the proportion of startups for which LLaMA 3 responds *Yes*, normalized by the total number of AI-tagged startups. The exposure of each occupation is finally computed as the average exposure of the DWAs associated with that occupation[2].

[2]It is also possible to weight each DWA by its importance to the occupation and compute a weighted average. We also implemented this approach using, as a proxy for DWA importance, the importance score of the corresponding broader Work Activity in O*NET. (In O*NET, Work Activities refer to a higher-level, hierarchical categorization of job behaviors, encompassing multiple DWAs). Results using this weighting scheme are qualitatively identical to those reported here.

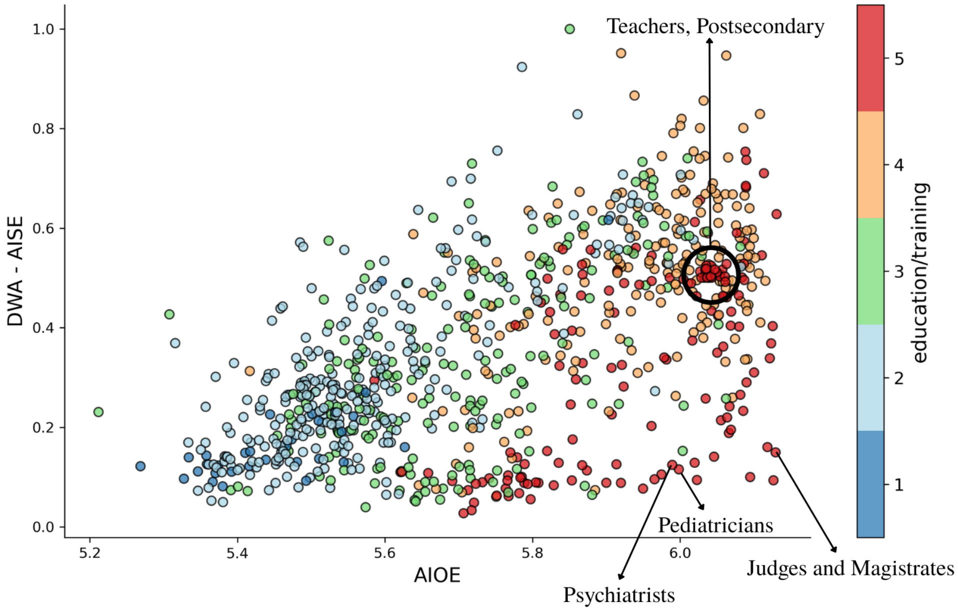

Supplementary Figure 17: **DWA Startups–based AISE vs AIOE.** Each point represents an occupation and the colors the corresponding job zone.

In Figure 17, we plot the DWA-based AISE index against the AIOE index, following the same approach as in Figure 1 of the main text. Overall, the same pattern observed in Figure 1 emerges: high-stake occupations, that require a high levels of responsibility, tend to cluster in the region of low AISE (i.e., less targeted by AI startups) and high AIOE (i.e., theoretically more exposed to AI). In this figure, this result appears even more pronounced, as the bottom-right region of the scatter plot contains only occupations from Job Zone 5—the zone requiring the highest levels of education and training.

There are, however, a few notable differences compared to the analysis in the main text. First, the correlation between AIOE and this DWA-based version of AISE is stronger than the correlation with the original AISE based on O*NET short descriptions. This is largely due to the fact that average exposure scores are generally higher in the DWA-based approach. As mentioned above, many occupations include several DWAs that are easily automatable by AI, which increases their overall exposure scores.

Second, and interestingly, most of the Job Zone 5 occupations for which the DWA-based AISE score is significantly higher than the one based on short descriptions are postsecondary teaching professions (highlighted with a circle in the figure). This effect arises because O*NET's short descriptions for teaching occupations often capture only their primary function. For example, the essential task mentioned in the description for *Economics Teachers, Postsecondary* is simply *Teach courses in economics.* In contrast, when using DWAs, the core teaching activity is just one among many activities that are highly targeted by AI startup— such as *Compile specialized bibliographies or lists of materials* or *Select educational materials or equipment.*

This represents a clear example of the conceptual difference between the two approaches and illustrates both their strengths and limitations: one focuses on the broader context and the intellectual and social role of the occupation, while the other focuses on the specific activities that make up the job. In other words, one perspective evaluates how much the startup ecosystem targets or interacts with the societal role of teachers, while the other assesses the extent to which startups are automating specific tasks.

Even though the differences are highly evident only for some occupations and the main conclusions obtained with the DWAs remain consistent with those of the main paper, these differences are nonetheless analytically interesting and will be the focus of future research.

References

Acemoglu, D. and Autor, D. (2011). Skills, tasks and technologies: Implications for employment and earnings. In *Handbook of labor economics*, volume 4, pages 1043–1171. Elsevier.

Acemoglu, D. and Restrepo, P. (2020). Robots and jobs: Evidence from US labor markets. *Journal of Political Economy*, 128(6):2188–2244.

Barbieri, L., Mussida, C., Piva, M., and Vivarelli, M. (2020). Testing the employment and skill impact of new technologies. *Handbook of labor, human resources and population economics*, pages 1–27.

Eloundou, T., Manning, S., Mishkin, P., and Rock, D. (2024). GPTs are GPTs: Labor market impact potential of LLMs. *Science*, 384(6702):1306–1308.

Felten, E., Raj, M., and Seamans, R. (2021). Occupational, industry, and geographic exposure to artificial intelligence: A novel dataset and its potential uses. *Strategic Management Journal*, 42(12):2195–2217.

Felten, E. W., Raj, M., and Seamans, R. (2018). A method to link advances in artificial intelligence to occupational abilities. In *AEA Papers and Proceedings*, volume 108, pages 54–57. American Economic Association.

Graetz, G. and Michaels, G. (2018). Robots at work. *Review of Economics and Statistics*, 100(5):753–768.

Pizzinelli, C., Panton, A. J., Tavares, M. M. M., Cazzaniga, M., and Li, L. (2023). *Labor market exposure to AI: Cross-country differences and distributional implications*. International Monetary Fund.

Soori, M., Arezoo, B., and Dastres, R. (2023). Artificial intelligence, machine learning and deep learning in advanced robotics, a review. *Cognitive Robotics*, 3:54–70.
